# Icaritin-curcumol activates CD8^+^ T cells through regulation of gut microbiota and the DNMT1/IGFBP2 axis to suppress the development of prostate cancer

**DOI:** 10.1186/s13046-024-03063-2

**Published:** 2024-05-23

**Authors:** Wenjing Xu, Yingqiu Li, Lumei Liu, Jing Xie, Zongren Hu, Shida Kuang, Xinying Fu, Bonan Li, Tiansong Sun, Congxu Zhu, Qinghu He, Wen Sheng

**Affiliations:** 1 Department of Dermatology, The First Affiliated Hospital of Hunan University of Chinese Medicine, Changsha, 410021 China; 2 Medical School, Hunan University of Chinese Medicine, Changsha, 410208 China; 3 School of Integrated Chinese and Western Medicine, Hunan University of Chinese Medicine, Changsha, 410208 China; 4 Andrology Laboratory, Hunan University of Chinese Medicine, Changsha, 410208 China; 5 School of Traditional Chinese Medicine, Hunan University of Medicine, No. 492 Jinxi South Road, Huaihua, 418000 China; 6 School of Rehabilitation Medicine and Health Care, Hunan University of Medicine, No. 492 Jinxi South Road, Huaihua, 418000 China; 7 School of Traditional Chinese Medicine, Hunan University of Chinese Medicine, Changsha, 410208 China

**Keywords:** Icariin-curcumol, Gut microbiota, Metabolism, Tumor immune microenvironment, Prostate cancer

## Abstract

**Background:**

Prostate cancer (PCa) incidence and mortality rates are rising. Our previous research has shown that the combination of icariin (ICA) and curcumol (CUR) induced autophagy and ferroptosis in PCa cells, and altered lipid metabolism. We aimed to further explore the effects of the combination of ICA and CUR on gut microbiota, metabolism, and immunity in PCa.

**Methods:**

A mouse subcutaneous RM-1 cell tumor model was established. 16 S rRNA sequencing was performed to detect changes in fecal gut microbiota. SCFAs in mouse feces, and the effect of ICA-CUR on T-cell immunity, IGFBP2, and DNMT1 were examined. Fecal microbiota transplantation (FMT) was conducted to explore the mechanism of ICA-CUR. Si-IGFBP2 and si/oe-DNMT1 were transfected into RM-1 and DU145 cells, and the cells were treated with ICA-CUR to investigate the mechanism of ICA-CUR on PCa development.

**Results:**

After treatment with ICA-CUR, there was a decrease in tumor volume and weight, accompanied by changes in gut microbiota. ICA-CUR affected SCFAs and DNMT1/IGFBP2/EGFR/STAT3/PD-L1 pathway. ICA-CUR increased the positive rates of CD3^+^CD8^+^IFN-γ, CD3^+^CD8^+^Ki67 cells, and the levels of IFN-γ and IFN-α in the serum. After FMT (with donors from the ICA-CUR group), tumor volume and weight were decreased. SCFAs promote tumor development and the expression of IGFBP2. In vitro, DNMT1/IGFBP2 promotes cell migration and proliferation. ICA-CUR inhibits the expression of DNMT1/IGFBP2.

**Conclusions:**

ICA-CUR mediates the interaction between gut microbiota and the DNMT1/IGFBP2 axis to inhibit the progression of PCa by regulating immune response and metabolism, suggesting a potential therapeutic strategy for PCa.

**Supplementary Information:**

The online version contains supplementary material available at 10.1186/s13046-024-03063-2.

## Introduction

Prostate cancer (PCa) is a malignant tumor affecting men and is an important cause of the global increase in male mortality [1]. The causes of PCa involve multiple factors [2]. Even with second-generation androgen receptor axis inhibitors as the primary treatment option, most metastatic cases progress to castration-resistant PCa after the initial treatment response with a poor prognostic outcome [3]. Therefore, new treatments are required to enhance the prospects for PCa patients.

Recent studies have shown that altered microbiota composition (ecological dysbiosis) may crucially contribute to the occurrence, development, and prognosis of PCa [4]. Gut microbiota is mainly responsible for maintaining the balance between host defense and immune tolerance, and its imbalance is related to various changes in the immune system [5]. Terrisse S et al. demonstrated the potential clinical application value of reversing intestinal ecological disorder and repairing acquired immune deficiency in PCa patients [6]. Immunotherapy offers a promising modality for instigating antitumor immunity in PCa, following impetus from advanced molecular diagnostic tools and discoveries in immune mechanisms [7]. It was reported that the immune system and gut microbiota could determine the efficacy of androgen deprivation therapy for PCa [6]. Notably, one of the main modes of gut microbiota modulation of antitumor immunity is through metabolites, which are small molecules that can diffuse from their initial location in the gut and influence local and systemic antitumor-immune responses [8]. Matsushita M et al. reported that gut microbiota-derived short-chain fatty acids (SCFAs) facilitated the expansion of PCa via insulin-like growth factor binding protein 2 (IGFBP2)/IGFBP3 signaling [9]. In addition, the unique characteristics of PCa cell metabolism, as well as the activation of specific metabolic pathways, drive the use of metabolic inhibitors [10]. Therefore, exploring the mechanisms of gut microbiota, metabolism and immunity in PCa is conducive to assisting with PCa treatment.

Icariin (ICA) is a natural pentenyl flavonoid derived from Epimedium [11], which displays anti-tumor properties against various cancers including PCa [12]. ICA has been reported to inhibit the development of PCa neuroendocrine differentiation [13]. Additionally, ICA hindered the progression of PCa by downregulating the serum adipokines triggered by high-fat diets [14]. Curcumol (CUR) is a hydrogenated austenitic compound with a hemiketal [15]. Our previous studies have shown that CUR regulates the PDK1/AKT/mTOR pathway through miR-9, affecting PCa development [16]. Furthermore, CUR suppressed PCa growth through the miR-125a/signal transducer and activator of transcription 3 (STAT3) axis [17]. For the combination of ICA and CUR, we have also conducted a preliminary study. Our previous results showed that ICA and CUR jointly regulated the miR-7/mTOR/SREBP1 pathway, induced autophagy and ferroptosis in PCa cells, and altered lipid metabolism [18]. The combination of ICA and CUR has not been reported to have an impact on the gut microbiota, metabolism, and immune system in PCa.

DNA Methyltransferase 1 (DNMT1), a member of the DNA methyltransferase family, is highly expressed in various cancers [19, 20]. High expression of DNMT1 promotes the proliferation and migration of PCa cells [21]. It has been reported that ICA inhibits the expression of DNMT1 in oral squamous cell carcinoma [22]. CUR has been reported to inhibit the expression of DNMT1 in choriocarcinoma stem-like cells [23]. IGFBP2 occupies a pivotal position within the IGFBP family and exerts significant oncogenic effects in diverse types of human cancers [24]. It has been reported that IGFBP2 is overexpressed in prostatic intraepithelial neoplasia and invasive cancer and serves as a marker for PCa [25]. However, it is unclear whether the combination of ICA and CUR directly regulates the expression of DNMT1 or IGFBP2. Programmed Death-Ligand 1 (PD-L1) is an inhibitory receptor expressed by T cells that suppress the activation of immune T cells [26, 27]. IGFBP2 regulates PD-L1 expression through the activation of the EGFR-STAT3 signaling pathway, and it may serve as a PD-L1 regulator for melanoma [28]. Based on these studies, we speculate that the combination of ICA and CUR directly targets the DNMT1/IGFBP2 axis to affect cancer cell development and the level of PD-L1 to influence the differentiation of immune T cells, thereby affecting cancer cell development. Furthermore, SCFAs facilitated the expansion of PCa via IGFBP2/IGFBP3 signaling [9]. SCFAs may be involved in post-translational modifications such as DNA methylation by DNMT1 [29]. Therefore, we hypothesize that the combination of ICA and CUR could intervene in the gut microbiota-SCFAs, and SCFAs may influence the levels of IGFBP2 in circulation, thereby affecting the EGFR/STAT3/PD-L1 pathway and regulating the differentiation of immune T cells.

In this study, we will investigate the mechanisms of action of ICA and CUR in PCa. Our investigation may provide new therapeutic avenues for managing PCa, as well as provide molecular insights into the mechanisms of PCa progression.

## Materials and methods

### Establishment of mouse subcutaneous RM-1 cell tumor model

All animal experiments were approved by the Ethics Committee of Hunan University of Chinese Medicine (LLBH-202303240004). Male C57BL/6 mice at the age of six weeks were procured from SJA Laboratory Animal Co. Ltd (Hunan, China). After one week of adaptive feeding, RM-1 cells (AW-CCM416, Abiowell) were injected subcutaneously. The number of cells injected into each nude mouse was 1 × 10^6^; the injection volume was 100 µL, and the injection location was the left armpit. After tumor implantation, tumor measurements were performed 2–3 times per week.

Experiment 1, after obvious subcutaneous tumor formation (8 days), the mice were divided into 5 groups (*n* = 6 mice/group): PCa, ICA, CUR, ICA + CUR, and Docetaxel (DOC) groups. In the PCa group, mice were administered the same volume of vehicle control as the other experimental groups. In the ICA group, ICA (10 mL/kg; B21576, shyuanye, China) was intraperitoneally injected five times a week [12]. In the CUR group, mice were injected intraperitoneally with CUR (10 mL/kg; B20342, shyuanye, China) every two days [16]. In the ICA + CUR group: mice were administered with an equal dose of ICA + CUR as other experimental groups. After injecting ICA, CUR should be injected into the mice immediately. In the DOC group, mice were injected with DOC (10 mL/kg; S61817, shyuanye, China) intraperitoneally once a week as a positive control [30].

In experiment 2, mice were divided into 2 groups (*n* = 6 mice/group): ICA + CUR and ICA + CUR + SCFAs groups. In the ICA + CUR group, mice were injected with ICA + CUR. The mice were provided with water for normal feeding. In the ICA + CUR + SCFAs group, mice were injected with ICA + CUR. The mice’s drinking water was supplemented with a mixture of SCFAs: 67.5 mmol/L sodium acetate (S434883, Aladdin, China), 40 mmol/L sodium butyrate (303410, Sigma, USA), and 25.9 mmol/L sodium propionate (P1880, Sigma, USA) [9].

In experiment 3, mice were divided into 4 groups (*n* = 6 mice/group): ICA + CUR + IgG, ICA + CUR + SCFAs + IgG, ICA + CUR + anti-IGFBP2, and ICA + CUR + SCFA + anti-IGFBP2 groups. In the ICA + CUR + IgG group, mice were injected with ICA + CUR, on day 7, mice were intraperitoneally injected with IgG. In the ICA + CUR + SCFAs + IgG group, mice were injected with ICA + CUR, and on day 7, mice were intraperitoneally injected with IgG (10 mg/kg; 6-001-F, and systems, USA) once a day. The mice’s drinking water was supplemented with a mixture of SCFAs. In the ICA + CUR + anti-IGFBP2 group, mice were injected with ICA + CUR, and on day 7, mice were intraperitoneally injected with anti-IGFBP2 (10 mL/kg). In the ICA + CUR + SCFAs + anti-IGFBP2 group, mice were injected with ICA + CUR, and on day 7, mice were intraperitoneally injected with anti-IGFBP2 (10 mL/kg) [31]. The mice’s drinking water was supplemented with a mixture of SCFAs.

Twenty-Five days after tumor implantation, the experiment was completed, and the mice were sacrificed by intraperitoneal injection of pentobarbital sodium (150 mg/kg) [32]. The tumor was excised for further experiments. Stool and serum samples were collected for 16 S rRNA sequencing, metabolic analyses, and other analyses.

### Fecal microbiota transplantation (FMT)

For FMT, after 3 days of antibiotic-containing water was administered to the mice, they were given normal water for 3 days. In the PCa or ICA + CUR groups, freshly excreted feces were collected and then mixed with sterile water. To remove solid impurities, a 70 μm nylon filter sieve was used. Oral injection of fecal bacterial suspension was performed via a tube-feeding needle at a rate of 200 µL/mouse/day (from the fourth day until the death of the mice) [33]. They were grouped into the FMT-PCa and FMT-PCa-ICA + CUR groups and five mice per group. In the PCa group, mice were administered the same volume of corn oil as the other experimental groups. In the FMT-PCa group, mice were administered the corn oil. In the FMT-PCa-ICA + CUR group, mice were injected with the same volume of ICA + CUR as the FMT-PCa group.

### Magnetic bead sorting of CD3^+^CD8^+^ T cells

A complete culture medium (2 mL) was taken to moisten a 40 μm cell strainer before placing the mouse spleen. The mouse spleen was cut with ophthalmic scissors and ground by pressing the bottom of the piston of a 5 mL syringe. The strainer was rinsed with 10 mL of RPMI-1640 complete medium (C3010-0500, Viva Cell). Cell precipitation was achieved by centrifugation at 1500 rpm. The supernatant was removed and 5 mL of red cell lysate was added, which was cleaved at room temperature and centrifuged to precipitate the cells. The supernatant was removed, and the cells were resuspended in 6 mL PBS and 1 mL buffer solution. Then 1.3 × 10^7^ cells were counted. After the cells were suspended in 120 µL precooled buffer solution, 30 µL Biotin-Antibody Cocktail was added to the mixture, and the cells were incubated in the refrigerator. One milliliter of the buffer solution was added and centrifuged to collect the precipitates. A buffer solution (120 µL) was added for re-suspension, and then 60 µL Anti-Biotin MicroBeads were added to cells. The cells were placed in a refrigerator for incubation. The LS column was balanced on a magnetic rack with a buffer solution for equilibration. Subsequently, the aforementioned cells were loaded onto the LS column. After cleaning the LC column with buffer solution, the passed cells were collected for follow-up experiments (approximately 5.7 × 10^6^ cells were counted after sorting). 1 × 10^5^ cells /100 µL were taken into EP tubes, and washed with 1 mL PBS. After centrifuging at 1500 rpm, the cell pellet was retained. This step was once more. After resuspending the cell pellet in 100µL of basal culture medium, the corresponding antibody CD3-FITC (11-0032-82, eBioscience) was added, mixed, and incubated (to distinguish negative at this time). After PBS washing of the cells, they were centrifuged and the cell pellets were retained. Cells were precipitated in a basal medium and analyzed using a flow cytometer (A00-1-1102, Beckman).

### Cell culture and treatment

Mouse PCa cells RM-1 and human PCa cells DU145 (AW-CCH043, Abiowell) were cultured in DMEM containing 10% FBS and 1% penicillin/streptomycin at 37℃ and 5% CO_2_ in a saturated humidity incubator.

In experiment 1, RM-1 and DU145 cells were grouped into the Control, ICA, CUR, and ICA + CUR groups. In the Control group, cells were cultured normally. In the ICA group, cells were treated with 30 µM ICA (N1705, APExBIO) for 48 h [34]. In the CUR group, cells were treated with 50 µg/mL CUR (HY- N0104, MCE) for 48 h [16]. In the ICA + CUR group, cells were treated with 30 µM ICA and 50 µg/mL CUR.

In experiment 2, RM-1 cells with CD8^+^ T cells were co-cultured. Then they were divided into RM-1 + T cells, ICA + T cells, CUR + T cells, and ICA + CUR + T cells groups. In the RM-1 + T cell group, cells were co-cultured with CD8^+^ T cells. In the ICA + T cell group, cells were exposed to 30 µM ICA for 48 h, followed by co-culture with CD8^+^ T lymphocytes. In the CUR + T cell group, cells were exposed to 50 µg/mL CUR for 48 h, followed by co-culture with CD8^+^ T lymphocytes. In the ICA + CUR + T cell group, cells were treated with 30 µM ICA and 50 µg/mL CUR for 48 h, followed by co-culture with CD8^+^ T lymphocytes.

In experiment 3, the RM-1 and DU145 cells were divided into Control, ICR + CUR, ICR + CUR + oe-NC, ICR + CUR + oe-DNMT1, ICR + CUR + oe-DNMT1 + siNC, ICR + CUR + oe-DNMT1 + si-IGFBP2 groups. In the Control group, cells were cultured under normal conditions. In the ICR + CUR group, cells were exposed to 30 µM ICA and 50 µg/mL CUR. In the ICR + CUR + oe-NC group, cells were treated with 30 µM ICA and 50 µg/mL CUR, then transfected with oe-NC. In the ICR + CUR + oe-DNMT1 group, cells were treated with 30 µM ICA and 50 µg/mL CUR, then transfected with oe-DNMT1. In the ICR + CUR + oe-DNMT1 + si-NC group, cells were treated with 30 µM ICA and 50 µg/mL CUR, then transfected with oe-DNMT1 + si-NC. In the ICR + CUR + oe-DNMT1 + si-IGFBP2 group, cells were treated with 30 µM ICA and 50 µg/mL CUR, then transfected with oe-DNMT1 + si-IGFBP2.

In experiment 4, the RM-1 and DU145 cells were divided into si-NC, si-DNMT1, si-DNMT1 + oe-NC, and si-DNMT1 + oe-IGFBP2 groups. In the si-NC group, cells transfected with si-NC. In the si-DNMT1 group, cells were transfected with si-DNMT1. In the si-DNMT1 + oe-NC group, cells were transfected with si-DNMT1 + oe-NC. In the si-DNMT1 + oe-IGFBP2 group, cells were transfected with si-DNMT1 + oe-IGFBP2.

In experiment 5, RM-1 and DU145 cells were grouped into the Control, Acetate, Propionate, Butyrate, and SCFAs groups. In the Control group, cells were cultured normally. In the Acetate group, the cells were treated with acetate for 72 h. In the Propionate group, cells were treated with propionate for 72 h. In the Butyrate group, cells were treated with butyrate for 72 h. In the SCFAs group, cells were treated with acetate, propionate, and butyrate together for 72 h.

### 16 S rRNA sequencing

Fecal samples from mice in the PCa, ICA, CUR, and ICA + CUR groups were collected to assess changes in microbial diversity. To obtain raw data, the Illumina NovaSeq PE250 was used for 16 S amplicon sequencing. DADA2 was invoked to denoise the raw data using the Qiime 2 analysis process. Denoised sequences were de-redundant to obtain the feature information directly. Species annotation was performed for each ASV sequence, and the species composition in the samples was measured by comparing species databases.

### Detection of SCFAs

Fecal samples from mice in the PCa, ICA, CUR, and ICA + CUR groups were collected to detect changes in SCFAs (acetic, propionic, isobutyric, butyric, isovaleric, and valeric acid). An appropriate amount of feces (50 mg-100 mg) with magnetic beads and 300 µL saline (including 37.3 µg/mL d7 isobutyric acid) was homogenized at 60 Hz for 60 s. Supernatants were centrifuged at 4℃, and 200 µL was removed, acidified by adding 10 µL of 5 M HCl, and vortexed. Anhydrous ether (200 µL) was used for extraction, vortexed, and centrifuged at 4℃. The collected supernatant was subjected to analysis using an Agilent 7890B-5977B gas chromatograph.

### Immunofluorescence (IF)

To assess Ki67 expression in the mouse tumors, The sections were immersed in a solution of sodium borohydride solution, soaked in 75% ethanol solution, and then placed in Sudan Black staining solution. The sections were incubated in a 5% BSA solution. Appropriate dilutions of primary antibody Ki67 (ab16667, 1:100, Abcam) were added dropwise overnight at 4℃. 50–100 µL CoraLite488-conjugated Affinipure Goat Anti-Rabbit IgG (H + L) (SA00013-2, Proteintech) fluorescent antibody was incubated. DAPI was used to stain nuclei. The sections were sealed using buffered glycerol and subsequently examined using a fluorescence microscope.

### Enzyme-linked immunosorbent assay (ELISA)

According to the kit’s instructions, ELISA was utilized to evaluate levels of IGFBP2 (CSB-E04589m, CUSABIO), interferon-γ (IFN-γ) (KE10001, Proteintech), interferon-α (IFN-α) (MFNAS0, R&D Systems), Perforin (Cbic-E13429m, CUSABIO), Granzyme A (CSB-E08717m, CUSABIO, China) and Granzyme B (CSB-E08720m, CUSABIO, China).

### Western blot (WB)

To assess PD-L1, IGFBP2, DNMT1, EGFR, STAT3, p-EGFR, and p-STAT3 protein expression levels, the total proteins were first extracted using RIPA (#P0013B, Beyotime, China), and protein quantification was performed using the BCA protein assay kit (BL521A, Biosharp, China). Protein was mixed with SDS-PAGE loading buffer (AWB0055, Abiowell, China), and adsorbed on Nitrocellulose membrane. PD-L1 (28076-1-AP, 1:600, Proteintech), IGFBP2 (ab188200, 1:1000, Abcam), DNMT1 (AWA44628, 1:1000, Abiowell), EGFR (ab52894, 1:5000, Abcam), STAT3 (10253-2-AP, 1:2000, Proteintech), p-EGFR (ab40815, 1:1000, Abcam), p-STAT3 (ab76315, 1:5000, Abcam), and β-actin (28076-1-AP, 1:600, Proteintech) were incubated overnight at 4℃. HRP goat anti-mouse/rabbit IgG was added. An ECL chemiluminescence solution was used for color development. β-actin was used as an internal reference.

### Flow cytometry (FCM)

A complete culture medium (2 mL) was taken to moisten a 40 μm cell strainer before placing the tumor. The tumor was cut with ophthalmic scissors and ground by pressing the bottom of the piston of a 5 mL syringe. The rest of the operation is as described above for magnetic bead sorting. RM-1 and DU145 cells were resuspended in 500 µL of 10% FBS 1640, added 1µL of Cell Stimulation Cocktail (plus protein transport inhibitors), and incubated at 37℃. The cells were resuspended in 500 µL 10% FBS 1640, and 1uL of Cell Stimulation Cocktail (plus protein transport inhibitors) was added and then stimulated at 37℃. After collecting the cells and centrifuging, the supernatant was discarded. Then, 1 mL of 0.5% BSA-PBS was added to wash the cells once, followed by centrifugation, and the supernatant was discarded. The corresponding antibodies CD3-FITC (11-0032-82, eBioscience, USA) and CD8-APC (17-0081-82, eBioscience, USA) were incubated, and light was avoided. Cells were washed with PBS. Next, the cells were resuspended in 500 µL of Intracellular Fixation buffer and fixed. After centrifuging and discarding the supernatant, the pellet was resuspended in 1× Permeabilization Buffer, followed by discarding the supernatant after centrifugation. Then, 100 µL of 1×Permeabilization Buffer was used to resuspend the cell pellet, and the corresponding antibody IFN-γ (12-7311-82, eBioscience, USA) or Ki67 (12-5698-82, eBioscience, USA) was added and mixed well. The mixture was incubated, protected from light, for 30 min. The cells were washed with 1 mL of 0.5% BSA-PBS, and the supernatant was discarded after centrifugation. Finally, the cells were resuspended in 150 µL of 0.5% BSA-PBS for FCM.

### Cell counting Kit-8 (CCK-8) assay

RM-1 and DU145 cells were digested with pancreatic enzymes (AWC0232, Abiowell) and inoculated at a density of 1 × 10^4^ cells/well. After adherent culture, 10 µL/well of CCK-8 (NU679, Dojindo) was added. The cells were incubated and analyzed using the Bio-Tek assay (MB-530, HEALES) at 0, 24, 48, and 72 h.

### Transwell assays

First, a transwell assay was performed to determine the migratory capacity of the cells. A complete medium (500 µL) was placed in the lower layer of a Transwell (3428, Corning). The treated cells were digested with trypsin, serum-free medium was added to resuspend the cells at 2 × 10^6^ cells/mL, and 100 µL of cells were added to each well. After being placed in incubation, the upper chamber was removed, and placed in a new well with PBS. Then the upper chamber was washed with PBS. The cells in the upper chamber were wiped with cotton swabs. The cells were fixed with 4% paraformaldehyde. Cells were stained with 0.1% crystal violet (AWC0333, Abiowell) and washed 5 times with water. Cells on the surface of the upper chamber were observed under an inverted microscope, and three fields of view were obtained. In the invasion assay, Matrigel-coated Transwell chambers (354262, BD) were used, following the same experimental procedures as the migration assay.

### Real-time quantitative polymerase chain reaction (RT-qPCR)

The expression levels of perforin, granzyme A, and granzyme B in sorted CD8^+^ T cells were detected. Total RNA was extracted from CD8^+^ T cells using Trizol (15596026, Thermo, America), and cDNA was obtained using a reverse transcription kit (CW2569, CowinBio, China). Finally, we performed specific amplification using the SYBR method on a fluorescence quantitative RT-PCR instrument (SPL0960, Thermo, USA). The primer information used in the experiment can be found in Table 1.

**Table 1:** Primer sequences

Gene	Sequence	Length
H-perforin	F GGGATTCCAGAGCCCAAGTG R GTGTGTCCACTGGGAAGGAG	230 bp
H-granzyme A	F AAGGGGGACGATGTGAAACC R GCACAACAAAGGGCTTCCAG	243 bp
H-granzyme B	F GGGCAGATGCAGACTTTCC R GGCCCCCAAGGTGACATTTA	198 bp
H-β-actin	F ACCCTGAAGTACCCCATCGAG R AGCACAGCCTGGATAGCAAC	224 bp
M-perforin	F TCTTGGTGGGACTTCAGCTT	148 bp
	R TGCTTGCATTCTGACCGAGT	
M-granzyme A	F AACCAGATGCCGAGTAGCAG R GAGGTCCCCTGCACAAATCA	169 bp
M-granzyme B	F GAAGCCAGGAGATGTGTGCT R GCACGTTTGGTCTTTGGGTC	183 bp
M-β-actin	F ACATCCGTAAAGACCTCTATGCC R TACTCCTGCTTGCTGATCCAC	223 bp

### Immunohistochemistry (IHC)

The expression of DNMT1 (bs-0678r, 1:200, Bioss) and IGFBP2 (ab188200, 1:200, Abcam) in mouse tumors was detected. Tumor sections were sequentially placed in xylene and gradient ethanol, followed by washing with distilled water. The sections were immersed in 0.01 M citrate buffer (pH 6.0) and boiled using an electric stove, then cooled to room temperature and removed from the slides, followed by washing with PBS. Next, 1% periodic acid was added to the sections and left at room temperature for 10 min, followed by rinsing with PBS. Diluted primary antibodies against DNMT1 and IGFBP2 were added to the sections and incubated overnight at 4℃. After washing the sections with PBS, 50 ~ 100 µL of HRP goat anti-rabbit-IgG was added and incubated at 37℃ for 30 min, followed by washing with PBS. Then, 50 ~ 100 µL of pre-made DAB working solution was added to the sections and incubated at room temperature, followed by washing with distilled water. The sections were counterstained with hematoxylin, rinsed with distilled water, and then blued in PBS. The sections were subsequently subjected to a graded series of alcohol solutions for dehydration. Finally, the sections were removed and placed in xylene, covered with neutral gum, and observed under a microscope.

### Co-immunoprecipitation (CO-IP)

To detect the interaction between DNMT1 and IGFBP2, we first extracted total protein from RM-1 and DU145 cells. The protein supernatant was then divided into 2 tubes and a certain amount of antibody DNMT1(AWA44628, 1:1000, Abiowell) and IGFBP2 (ab188200, 1:1000, Abcam) was added, followed by thorough mixing and incubation overnight at 4℃ with rotation. Next, Protein A/G agarose beads were taken and mixed with IP lysis buffer (AWB0144, Abiowell, China), then centrifuged to collect the precipitate. The sample was supplemented with the pre-treated Protein A/G agarose beads and gently shaken at 4℃. After immunoprecipitation, the mixture was centrifuged, and the bottom of the agarose bead tube was retained to remove the supernatant, followed by washing the agarose beads with 400 µL IP lysis buffer for 4 times, and retaining the final precipitate. In the agarose bead precipitate, 30 µL of IP lysis buffer was added, followed by mixing with 10 µL of 5× loading buffer. The mixture was boiled, and then quickly cooled in an ice box for subsequent WB analysis.

### Statistical analysis

GraphPad Prism 8.0 software was applied for statistical analysis. Data were expressed as mean ± standard deviation. Student’s *t*-test was used between the two groups, and one-way analysis of variance (ANOVA) was performed for inter-group comparison. A correlation analysis was performed using the Spearman correlation coefficient to examine the relationship among gut microbiota with the IGFBP2/EGFR/STAT3/PD-L1 pathway and immune-related indicators. *P* < 0.05 indicated that the difference was statistically significant.

## Results

### ICA-CUR inhibits the development of PCa and regulates gut microbiota and SCFAs

**Fig. 1:**
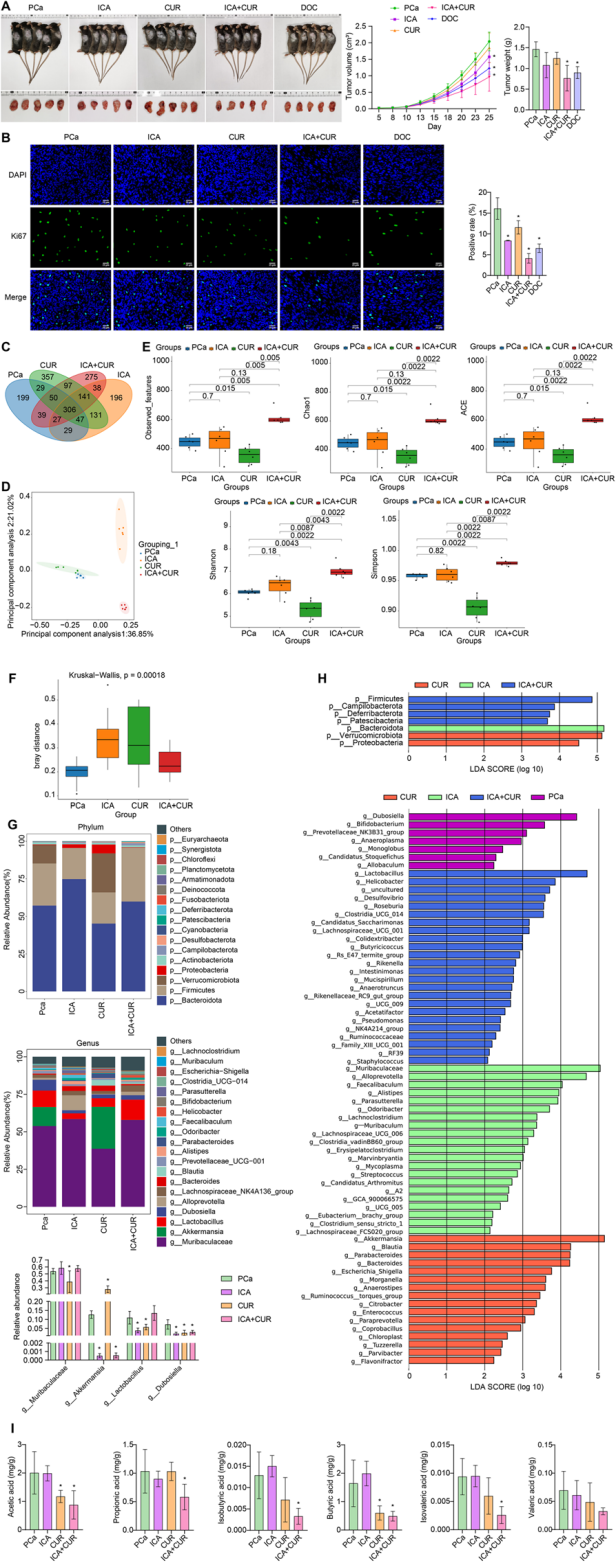
ICA-CUR inhibits the development of PCa and regulates gut microbiota and SCFAs. **A** Tumor imaging, volume, and weight measurements. **B** IF staining to examine changes in Ki67 expression in tumors. **C **Venn diagram analysis of differential OUTs among the different groups. **D** Principal component analysis plot. **E** α-diversity analysis (Observed OTUs, Simpson’s index, Shannon index, Chao1 index, PD whole tree). **F** β-diversity analysis. **G** Changes in the relative abundance of gut microbiota at the phylum and genus levels. **H** LefSe analysis of overall microbial structure differences at the phylum and genus levels. **I** Changes in SCFAs (acetic acid, propionic acid, isobutyric acid, butyric acid, isovaleric acid, and valeric acid) in mouse feces. *P < 0.05 vs. PCa

To investigate the effect of combined ICA and CUR treatment on PCa, a mouse subcutaneous RM-1 cell tumor model was first established. Subsequently, the mice were respectively treated with ICA, CUR, ICA-CUR, and DOC (positive control), and changes in tumor size were observed. As compared to the PCa group, tumor size and weight witnessed a decrease in the ICA, CUR, ICA + CUR, and DOC groups, with the most substantial reduction observed in the ICA + CUR group (Fig. 1A). Ki67 is a marker protein associated with cancer cell proliferation, expressed in various cancers, and used as a prognostic marker [35, 36]. Moreover, Ki67 expression levels were noted to be lower in the ICA, CUR, ICA + CUR, and DOC groups than those in the PCa group. Noticeably, the expression of Ki67 in the ICA + CUR group witnessed the most significant decline (Fig. 1B). The above results preliminarily indicated that the efficacy of ICA combined with CUR in treating PCa was significantly better than when ICA and CUR were used alone. The gut microbiota metabolizes the active ingredients of traditional Chinese medicine, which can help maintain intestinal health by balancing the microbial population [37]. Researchers have found that ICA is easily metabolized by gut microbiota or enzymes after oral administration [38]. Additionally, CUR may improve liver inflammation by influencing the gut microbiota [39]. Therefore, the changes in gut microbiota following treatment with ICA + CUR were further analyzed using 16 S rRNA sequencing. Figure 1C showed differences in OTU among groups. Compared to the PCa group, the number of gut microbiota in the ICA and ICA + CUR groups increased. Principal component analysis showed the degree of dispersion in each sample group. Compared to the PCa group, the microbial communities of the ICA and ICA + CUR group samples were significantly different (Fig. 1D). Besides, the alpha diversity (Observed, Chao1, ACE, Shannon, Simpson index) and β-diversity results of the mice showed that the gut microbiota in the ICA + CUR group was the most abundant, and there was a significant difference between the ICA + CUR group and the PCa group, indicating that the treatment of the ICA + CUR group may have an impact on the gut microbiota (Fig. 1E and F). Further investigation revealed changes in the gut microbiota of mice at the phylum and genus levels, as well as the overall structural differences at the phylum and genus levels. It was observed that after ICA + CUR treatment, the levels of *Akkermansia*, and *Dubosiella* within the gut of the mice significantly decreased at the genus level (Fig. 1G and H). Research reported that *Akkermansia* are important producers of SCFAs, primarily acetic and butyric acid [40]. Yuan et al. found that allicin could improve intestinal injury by affecting the production of acetic and propionic acid by *Dubosiella* [41]. In addition, the changes in SCFAs in the mouse feces showed a significant decrease in acetic acid, butyric acid, and propionic acid after ICA + CUR treatment (Fig. 1I). This result corresponded to the decrease at the genus level of *Akkermansia*, and *Dubosiella* after ICA + CUR treatment. Based on the above results, it was indicated that ICA + CUR inhibits the development of PCa and could regulate gut microbiota and SCFAs changes. Additionally, ICA + CUR may suppress the levels of SCFAs by inhibiting gut microbiota.

### ICA-CUR inhibits DNMT1/IGFBP2 pathway and activates cytotoxic effect of CD8^+^ T cell

**Fig. 2:**
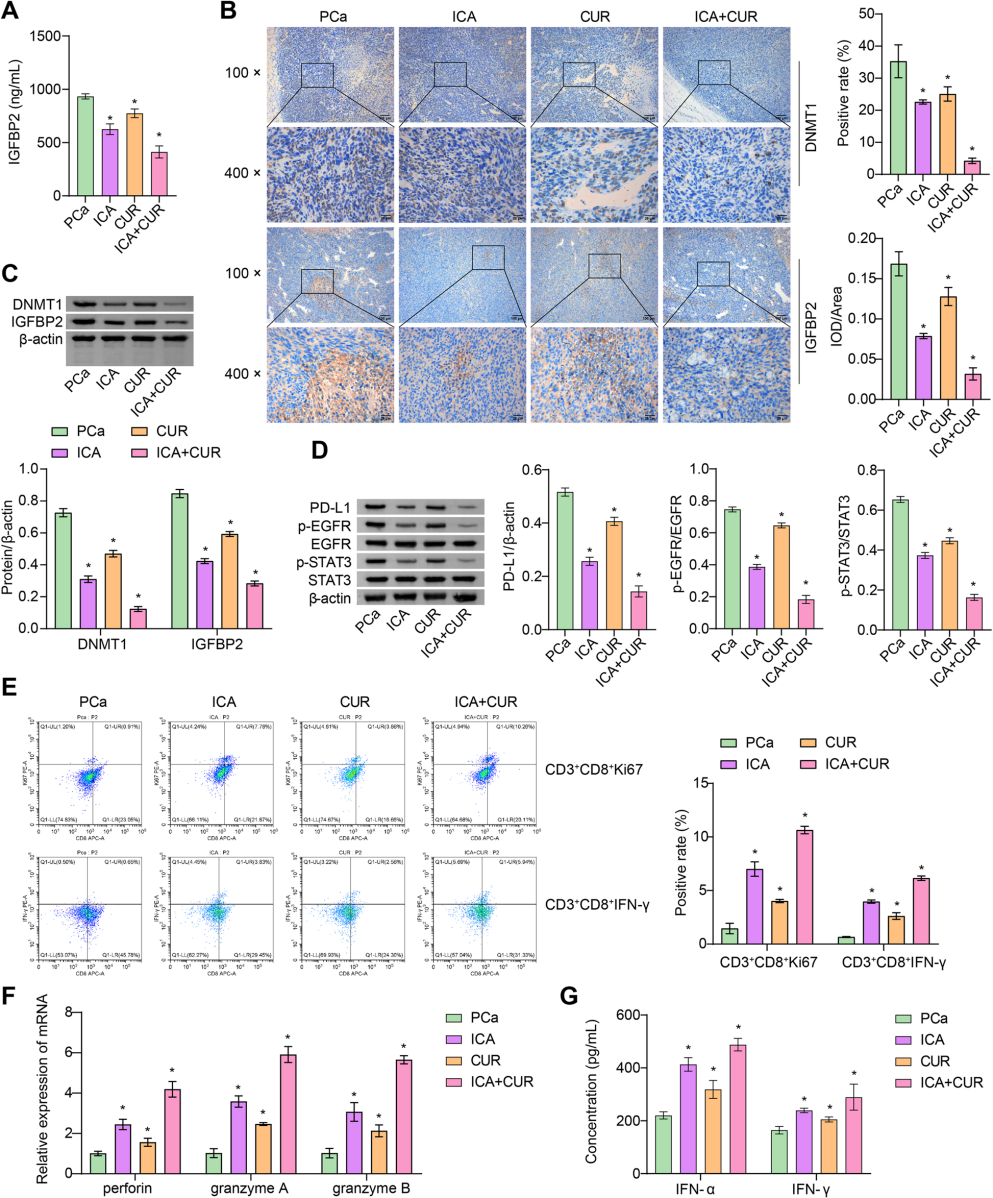
ICA-CUR inhibits the DNMT1/IGFBP2 pathway and activates the cytotoxic effect of CD8^+^ T cell. **A** The levels of IGFBP2 in serum were tested via ELISA. **B** The levels of DNMT1 and IGFBP2 in tumor tissues were tested via IHC (Magnification: ×100, scale bar = 100 μm; Magnification: ×400, scale bar = 25 μm). **C** The levels of DNMT1 and IGFBP2 in tumor tissues were detected by WB. **D** The protein changes of PD-L1, EGFR, STAT3, p-EGFR, and p-STAT3 in tumor tissues were detected by WB. **E** FCM was used to test the infiltration of CD8^+^ T cells (positivity of CD3^+^CD8^+^IFN-γ and CD3^+^CD8^+^Ki67 cells). **F** The levels of perforin, granzyme A, and B in sorted CD8^+^ T cells from tumor tissues were detected by RT-PCR. **G** The levels of IFN-γ and IFN-α in serum were tested via ELISA. *P < 0.05 vs. PCa

Next, the potential mechanisms through which ICA + CUR may influence PCa were investigated. Research reported that SCFAs can regulate host systemic and local prostate insulin-like growth factor 1, promoting PCa cell proliferation [9]. IGFBP2 is an important oncogene in various human cancers [24]. Therefore, the levels of IGFBP2 in the serum were first measured. Compared with the PCa group, serum IGFBP2 levels were decreased in the ICA, CUR, and ICA + CUR groups. Among them, the trend in the ICA + CUR group was more obvious (Fig. 2A). The expression of DNMT1 and IGFBP2 in the tumors of the ICA, CUR, and ICA + CUR groups was also decreased, but the ICA + CUR group exhibited the most significant decrease (Fig. 2B, C). In addition, the potential downstream expressions of IGFBP2, including PD-L1, p-EGFR, and p-STAT3 also significantly decreased in the tumors. Among them, the ICA + CUR group showed a more pronounced trend (Fig. 2D). These results preliminary indicated that ICA + CUR had an inhibitory effect on the DNMT1/IGFBP2 pathway. To determine whether ICA + CUR could induce an anti-tumor immune response, the infiltration of CD8^+^ T cells in the tumors was examined. The results showed that the positive rates of CD3^+^CD8^+^Ki67 cells and CD3^+^CD8^+^IFN-γ cells were lower in the PCa group, while they were increased after treatment with ICA, CUR, and ICA + CUR. The ICA + CUR group showed the most significant increase in positive rates. Furthermore, the increase in positive rates of CD3^+^CD8^+^Ki67 cells was more pronounced in the ICA + CUR group compared to CD3^+^CD8^+^IFN-γ cells (Fig. 2E). The study reported that CD8^+^ T cells release perforin, granzyme A, and B when exerting their cytotoxic effects [42]. IFN-α and IFN-β are effector factors that enhance CD8^+^ T cell responses [43, 44]. Therefore, the levels of perforin, granzyme A, and granzyme B in the sorted CD8^+^ T cells from mouse tumors were measured. Compared to the PCa group, the ICA, CUR, and ICA + CUR groups showed increased expression of perforin, granzyme A and B, with the ICA + CUR group showing the most significant trend (Fig. 2F). Similarly, the levels of serum IFN-γ and IFN-α escalated in the ICA, CUR, and ICA + CUR groups than in the PCa group. The changes in the ICA + CUR group were the most conspicuous (Fig. 2G). These results indicated that ICA-CUR activates the cytotoxic effect of CD8^+^ T cells, triggering an antitumor immune response, and suppressing the DNMT1/IGFBP2/EGFR/STAT3/PD-L1 axis.

### FMT from donors in the ICA + CUR group inhibits the development of PCa and activates the cytotoxic effects of CD8^+^ T cells

**Fig. 3:**
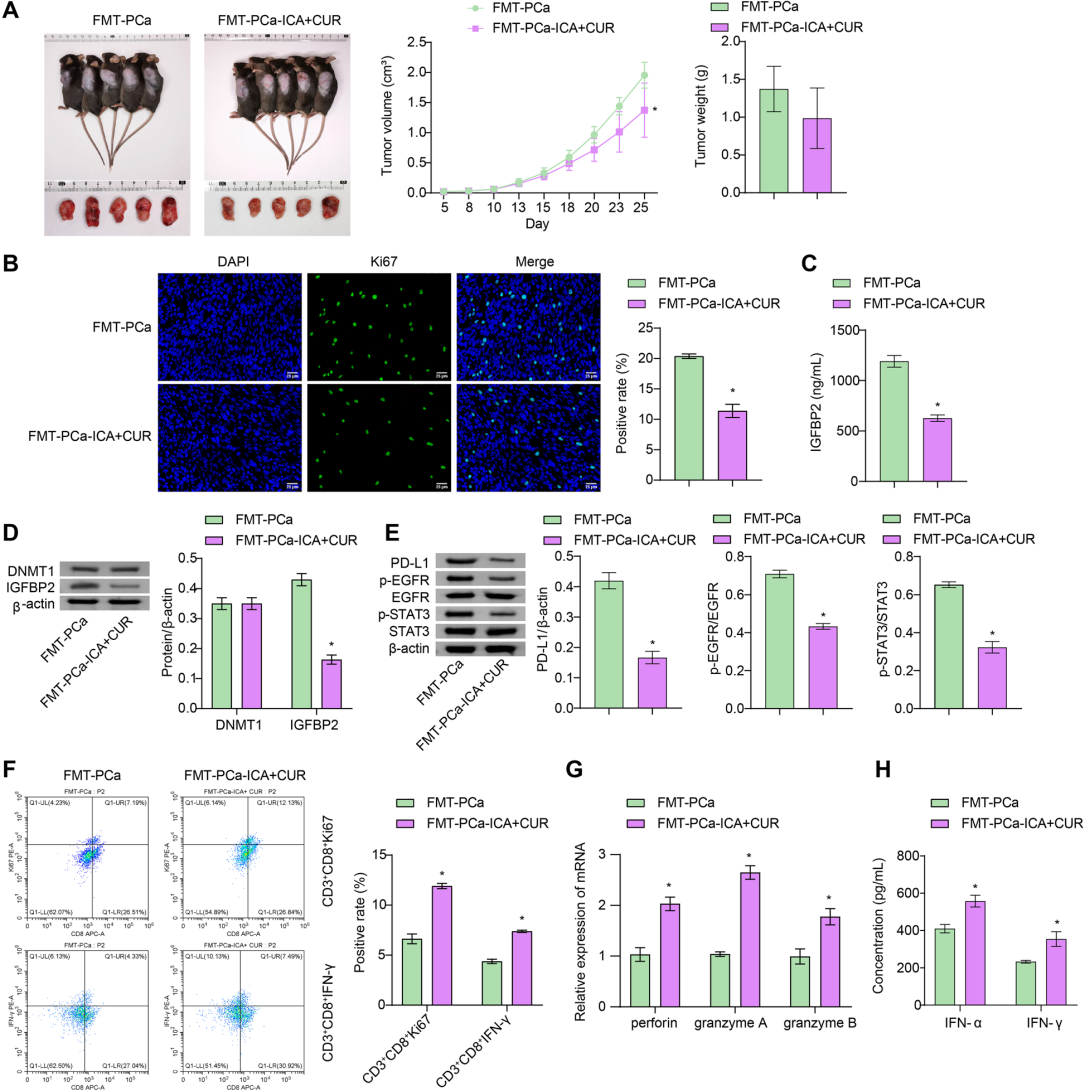
FMT from donors in the ICA-CUR treatment inhibits the development of PCa and activates the cytotoxic effects of CD8^+^ T cells. **A** Tumor imaging, volume, and weight measurements. **B** IF staining was utilized to examine changes in Ki67 expression in tumors (Magnification: ×400, scale bar = 25 μm). **C** The levels of IGFBP2 in serum were tested via ELISA. **D** The levels of DNMT1 and IGFBP2 in tumor tissues were detected by WB. **E** WB was used to detected the protein changes of PD-L1, EGFR, STAT3, p-EGFR, and p-STAT3 in tumor tissues. **F** The infiltration of CD8^+^ T cells in mouse tumor tissues (positivity of CD3^+^CD8^+^IFN-γ and CD3^+^CD8^+^Ki67 cells) was detected by FCM. **G** The expression of perforin, granzyme A, and B in sorted CD8^+^ T cells from tumor tissues was detected by RT-PCR. **H** The levels of IFN-γ and IFN-α in serum were tested via ELISA. *P < 0.05 vs. FMT-PCa

Additionally, the mice were treated with FMT (with donors from the PCa and ICA-CUR group). The results showed that after FMT (with donors from the ICA-CUR group), the tumor volume and weight of the mice were reduced (Fig. 3A). Moreover, compared to the FMT-PCa group, Ki67 expression in the FMT-PCa-ICA + CUR group was reduced (Fig. 3B). These results indicated that the ICA + CUR treatment-mediated gut microbiota inhibited tumor growth. Similarly, compared to the FMT-PCa group, serum IGFBP2 levels were decreased in the FMT-PCa-ICA + CUR group (Fig. 3C). Compared to the FMT-PCa group, the FMT-PCa-ICA + CUR group experienced a reduction in the expression of IGFBP2 in the tumor tissues, with no significant change in DNMT1. Besides, PD-L1, p-EGFR, and p-STAT3 protein expression were also decreased (Fig. 3D and E). The ICA + CUR treatment-mediated gut microbiota was found to inhibit the IGFBP2 pathway. The infiltration of CD8^+^ T cells in the mouse tumor was examined next. We found that compared to the FMT-PCa group, the FMT-PCa-ICA + CUR group had an increased positivity rate of CD3^+^CD8^+^Ki67 and CD3^+^CD8^+^IFN-γ cells (Fig. 3F). Additionally, compared to the FMT-PCa group, the FMT-PCa-ICA + CUR group showed significantly increased expression of perforin, granzyme A and B in CD8^+^ T cells, as well as serum IFN-γ and IFN-α levels, indicating that the ICA + CUR treatment-mediated gut microbiota activated the cytotoxic effect of CD8^+^ T cells (Fig. 3G and H). In conclusion, the ICA + CUR treatment-mediated gut microbiota inhibits the development of PCa and inflammation and activates the cytotoxic effects of CD8^+^ T cells.

### ICA-CUR inhibits tumor development and activates cytotoxic effects of CD8^+^ T cells by suppressing the SCFAs-IGFBP2 axis

**Fig. 4:**
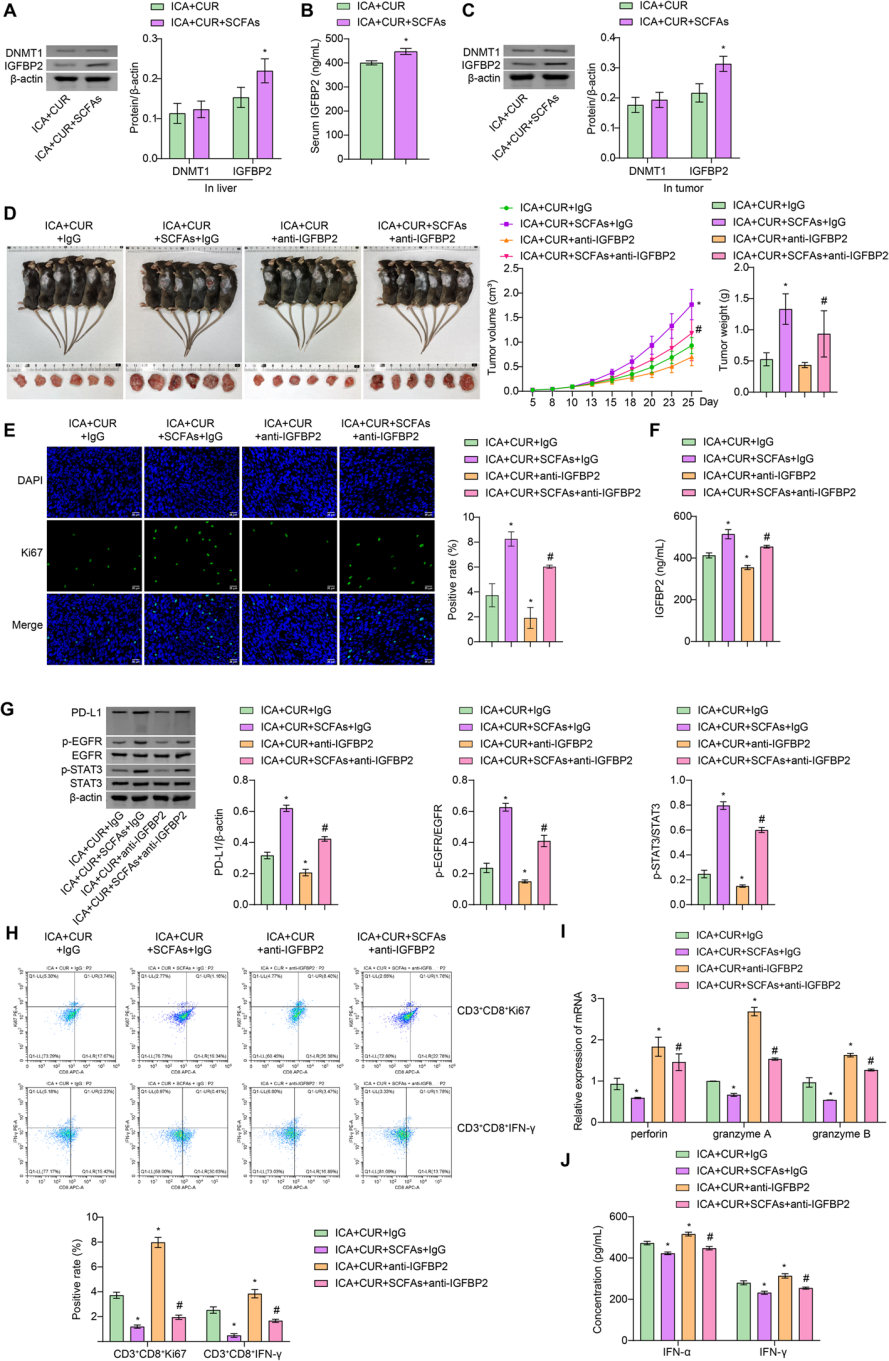
ICA-CUR inhibits tumor development and activates cytotoxic effects of CD8^+^ T cells by suppressing the SCFAs-IGFBP2 axis. **A** The levels of DNMT1 and IGFBP2 in tumor tissues were detected by WB. **B** The levels of IGFBP2 in serum were tested via ELISA. **C** The levels of DNMT1 and IGFBP2 in tumor tissues were detected by WB. *P < 0.05 vs. ICA + CUR. **D** Tumor imaging, volume, and weight measurements. **E** IF staining to examine changes in Ki67 expression in tumors (Magnification: ×400, scale bar = 25 μm). **F** ELISA was used to detect the levels of IGFBP2 in serum. **G** The protein changes of PD-L1, EGFR, STAT3, p-EGFR, and p-STAT3 in tumor tissues were detected by WB. **H** The infiltration of CD8^+^ T cells in mouse tumor tissues (positivity of CD3^+^CD8^+^IFN-γ and CD3^+^CD8^+^Ki67 cells) was detected by FCM. **I** The levels of perforin, granzyme A, and B in sorted CD8^+^ T cells from tumor tissues were detected by RT-PCR. **J** ELISA was utilized to detect the levels of IFN-γ and IFN-α in serum. *P < 0.05 vs. ICA + CUR + IgG, #*P* < 0.05 vs. ICA + CUR + anti-IGFBP2

To investigate the relationship between SCFAs and IGFBP2, the expression of DNMT1 and IGFBP2 in mouse liver was first examined. Compared to the ICA + CUR group, the ICA + CUR + SCFAs group showed a significant increase expression of IGFBP2 in the liver and tumor tissues, while DNMT1 expression showed no significant change. In addition, the levels of IGFBP2 in the serum were increased in the ICA + CUR + SCFAs group (Fig. 4A, B and C). These results indicated that SCFAs promoted the expression of IGFBP2. Further results showed that compared to the ICA + CUR + IgG group, mice treated with SCFAs exhibited a significant increase in the tumor volume and weight, and the expression of Ki67 in the tumor was also increased. Additionally, upon the addition of anti-IGFBP2 treatment to ICA + CUR + SCFAs + IgG, the tumor shrunk, Ki67 levels in the tumors decreased, and the tumor-promoting effect of SCFAs on tumor growth was weakened (Fig. 4D and E). Compared to the ICA + CUR + IgG group, the levels of IGFBP2 and its downstream target proteins including PD-L1, p-EGFR, and p-STAT3 in the ICA + CUR + SCFAs + IgG group were all increased. Upon inhibition of IGFBP2 expression, the expression of these markers was decreased (Fig. 4F and G). These results demonstrated that ICA + CUR inhibits tumor growth by regulating the SCFAs-IGFBP2 axis. In addition, compared to the ICA + CUR + IgG group, the ICA + CUR + SCFAs + IgG group showed a significantly lower positivity rate of CD3^+^CD8^+^Ki67 and CD3^+^CD8^+^IFN-γ cells. However, after anti-IGFBP2 treatment, the positivity rates of cells increased (Fig. 4H). Furthermore, compared to ICA + CUR + IgG, the levels of perforin, granzyme A, and granzyme B in CD8^+^ T cells, as well as IFN-γ and IFN-α in the serum, all decreased in the ICA + CUR + SCFAs + IgG group. However, the addition of anti-IGFBP2 reversed the effects of SCFAs, indicating that SCFAs weaken the cytotoxic effects of CD8^+^ T cells through IGFBP2 (Fig. 4I and J). Combining the above results, it was speculated that ICA + CUR inhibits the development of PCa by suppressing the SCFAs-IGFBP2 axis, and activates the cytotoxic effect of CD8^+^ T cells.

### ICA-CUR inhibits the development of PCa, the DNMT1/IGFBP2 pathway, and activates cytotoxic effects of CD8^+^ T cells in vitro

**Fig. 5:**
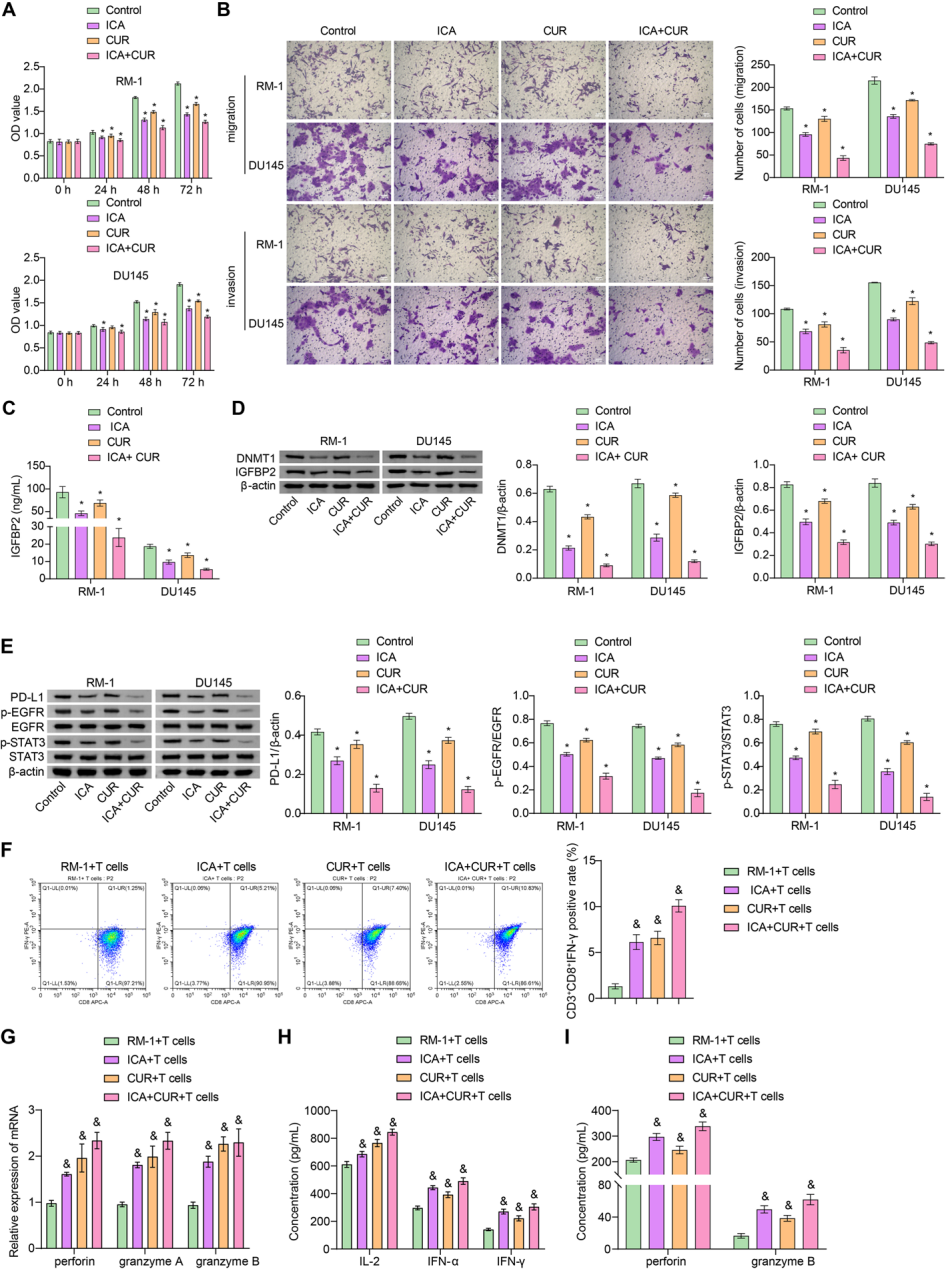
ICA-CUR inhibits the development of PCa, the DNMT1/IGFBP2 pathway, and activates cytotoxic effects of CD8^+^ T cells in vitro. **A** CCK-8 was utilized to assess the proliferation ability of cells. **B** Transwell was applied to measure the migration and invasion ability of cells. **C** The level of IGFBP2 in cells was tested via ELISA. **D** The levels of DNMT1 and IGFBP2 in cells were detected by WB. **E** WB was utilized to monitor protein changes of PD-L1, EGFR, STAT3, p-EGFR, and p-STAT3. *P < 0.05 vs. Control. **F** FCM analysis of CD8^+^ IFN-γ cells. **G** The expression of perforin, granzyme A, and B in sorted CD8^+^ T cells was detected by WB. **H** IL-2, IFN-γ, and IFN-α levels in supernatant were measured through ELISA. **I** Perforin and granzyme B levels in the supernatant were tested via ELISA. & *P* < 0.05 vs. RM-1 + T cells

In previous experiments, it has been demonstrated that ICA + CUR may mediate the development of PCa and T cell immunity through the gut microbiota-SCFAs-IGFBP2 pathway. In vivo experiments have shown that ICA-CUR inhibits the production of SCFAs. However, considering the relatively low overall content of isobutyric acid and isovaleric acid, the effects of acetate, propionate, butyrate, and SCFAs on PCa were first investigated in vitro. Compared to the Control group, there was no significant change in the cell viability of RM-1 and DU145 cells in the acetate, propionate, and butyrate groups, but the cell viability in the SCFAs group increased, indicating that SCFAs promote the proliferation of RM-1 and DU145 cells (Figure S1A). Compared to the Control group, the levels of IGFBP2 in RM-1 and DU145 cells were significantly increased in the acetate, propionate, butyrate, and SCFAs groups, while DNMT1 showed no significant change (Figure S1B-1 C). These results further confirm that SCFAs could directly promote the level of IGFBP2 and that SCFAs promote the progression of PCa. Next, the mechanism of action of ICA-CUR on PCa was further investigated through in vitro cell experiments. Given that ICA + CUR inhibits the expression of DNMT1 in vivo, and since the ICA + CUR treatment of FMT and direct treatment with SCFAs had no significant effect on the expression of DNMT1 in vivo, the investigation would continue to determine whether ICA + CUR directly affected the expression of DNMT1 in PCa cells in vitro. The results showed that compared to the control group, proliferation, migration, and invasion abilities in the ICA, CUR, and ICA + CUR groups were repressed, with the most significant trend observed in the ICA + CUR group, indicating that ICA + CUR inhibits the development of PCa (Fig. 5A and B). In addition, in comparison to the Control group, the levels of DNMT1, IGFBP2, and the downstream target proteins including PD-L1, p-EGFR, and p-STAT3 were all downregulated in the ICA, CUR, and ICA + CUR groups, with the most significant changes observed in the ICA + CUR group, indicating that ICA + CUR may directly regulate the DNMT1/IGFBP2 pathway (Fig. 5C, D and E). Additionally, magnetic bead separation resulted in a CD8^+^T-cells positive rate of 94.80% (Figure S2), followed by co-culturing RM-1 cells with CD8^+^T cells. Compared with the RM-1 + T cells group, positive rates of CD3^+^CD8^+^IFN-γ cells were increased, and the levels of perforin, granzyme A, and B were also increased in the cells, with the most significant elevation observed in the ICA + CUR + T cells group (Fig. 5F and G). IL-2, IFN-γ, IFN-α, perforin, and granzyme B levels in the cell supernatant were also increased, with the most obvious changes in the ICA + CUR + T cell group (Fig. 5H and I). These results indicated that ICA + CUR could activate the cytotoxic effect of CD8^+^ T cells.

### ICA-CUR inhibits the development of PCa and activates cytotoxic effects of CD8^+^ T cells through the inhibition of the DNMT1/IGFBP2 pathway

**Fig. 6:**
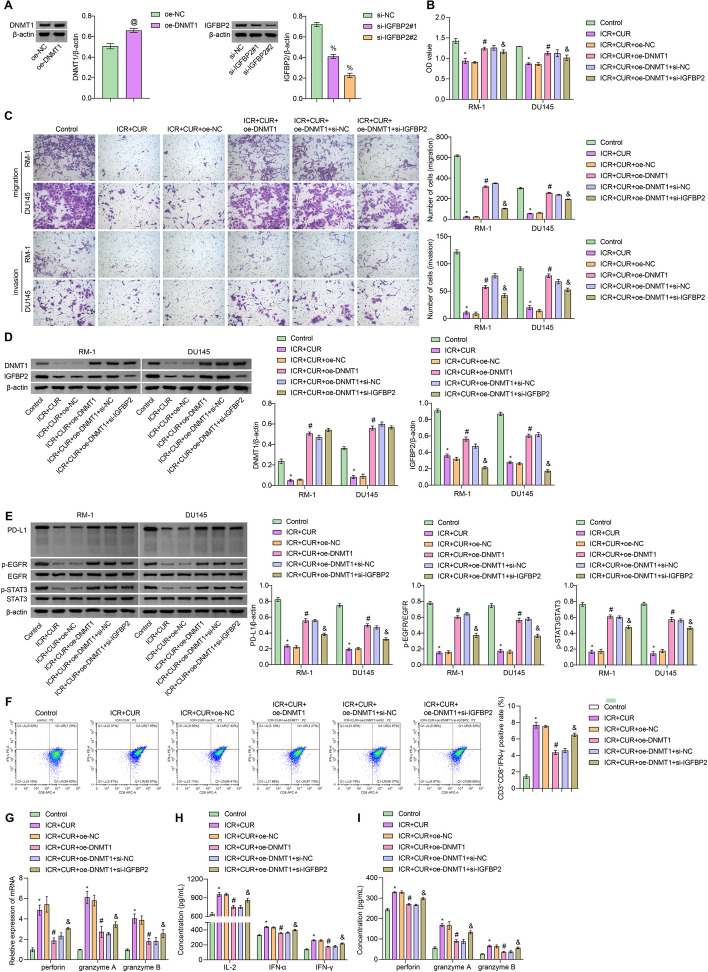
ICA-CUR inhibits the development of PCa and activates the cytotoxic effects of CD8^+^ T cells through the inhibition of the DNMT1/IGFBP2 pathway. **A** Transfection efficiency detection by WB. **B** CCK-8 was utilized to assess the proliferation ability of cells. **C** Cell migration and invasion ability detection by Transwell assay. **D** The levels of DNMT1 and IGFBP2 in cells were detected by WB. **E** WB was utilized to monitor protein changes of PD-L1, EGFR, STAT3, p-EGFR, and p-STAT3. **F** FCM analysis of CD3^+^CD8^+^IFN-γ cells. **G** The levels of perforin, granzyme A, and B in sorted CD8^+^ T cells were detected by RT-qPCR. **H** The levels of IL-2, IFN-γ, and IFN-α in serum. **I** perforin, granzyme A and B levels in the supernatant were tested via ELISA. *P < 0.05 vs. Control, # *P* < 0.05 vs. ICR + CUR + oe-NC, & *P* < 0.05 vs. ICR + CUR + oe-DNMT1 + si-NC

First, molecular docking analyses were performed for ICA and DNMT1, as well as CUR and DNMT1, to further confirm that ICA + CUR can directly act on DNMT1. The results showed successful docking between ICA and DNMT1, as well as CUR and DNMT1 molecules (Figure S3). RM-1 and DU145 cells were transfected with oe-DNMT1 and si-IGFBP2 plasmids, and the increased expression of DNMT1 and decreased expression of IGFBP2 indicated successful transfection, with si-IGFBP2#2 showing a higher transfection efficiency (Fig. 6A). Subsequent experiments were carried out using si-IGFBP2#2. Next, it was observed that compared to the Control group, the ICA + CUR group exhibited decreased proliferation, migration, and invasion abilities. In contrast, the proliferation, migration, and invasion abilities were increased in the ICA + CUR + oe-DNMT1 group compared to the ICA + CUR + oe-NC group. However, transfection with si-IGFBP2 weakened the effects of oe-DNMT1, resulting in decreased proliferation, migration, and invasion abilities (Fig. 6B and C). In addition, the ICA + CUR + oe-DNMT1 group showed increased protein expression of DNMT1, IGFBP2, and the downstream proteins compared to the ICA + CUR + oe-NC group. Interfering with the expression of IGFBP2 resulted in no significant change in DNMT1 expression, while the expression of IGFBP2, PD-L1, p-EGFR, and p-STAT3 decreased (Fig. 6D and E). In conclusion, it was speculated that ICA + CUR could inhibit the progression of PCa by inhibiting the DNMT1/IGFBP2 pathway in vitro. Next, RM-1 cells were co-cultured with CD8^+^ T cells, and it was found that compared to the Control group, the ICA + CUR group showed an increase in the positivity rate of CD3^+^CD8^+^ IFN-γ cells, elevated levels of perforin, granzyme A and B in the CD8^+^T cells and cell supernatant. The levels of these indicators in the ICA + CUR + oe-DNMT1 group were decreased compared to the ICA + CUR + oe-NC group, and transfection with si-IGFBP2 weakened the effect of oe-DNMT1 (Fig. 6F, G, H and I). These results indicated that in vitro, ICA + CUR could activate the cytotoxic effects of CD8^+^ T cells by inhibiting the DNMT1/IGFBP2 pathway.

### Inhibiting DNMT1 could suppress PCa development and activate the cytotoxic effects of CD8^+^ T cells by inhibiting the IGFBP2/EGFR/STAT3/PD-L1 pathway

**Fig. 7:**
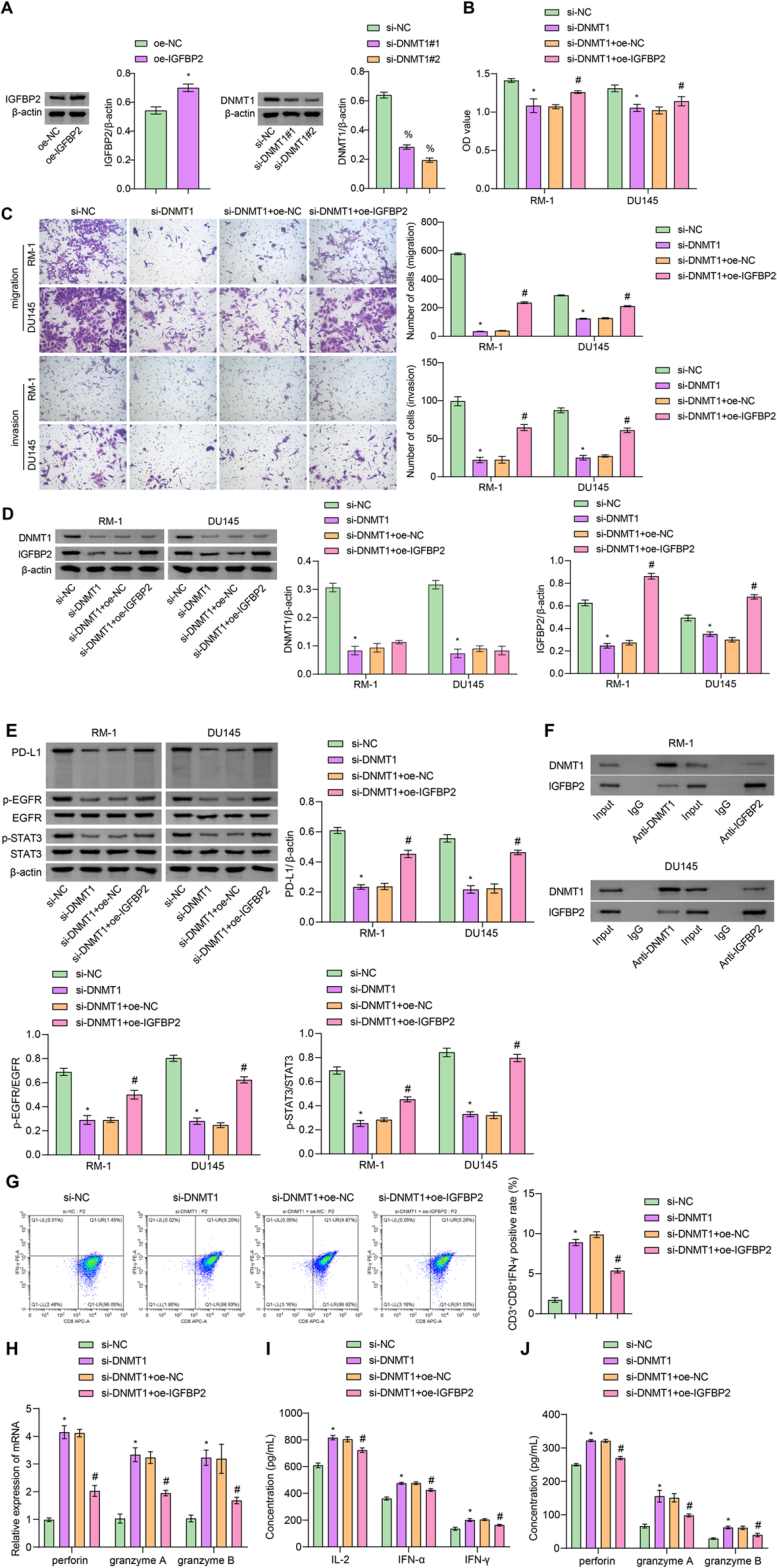
Inhibiting DNMT1 could suppress PCa development and activate the cytotoxic effects of CD8^+^ T cells by inhibiting the IGFBP2/EGFR/STAT3/PD-L1 pathway. **A** Transfection efficiency detection by WB. **B** CCK-8 was utilized to assess the proliferation ability of cells. **C** Cell migration and invasion ability detection by Transwell assay. **D** The levels of DNMT1 and IGFBP2 were detected by WB. **E**, **F** WB was utilized to monitor protein changes of PD-L1, EGFR, STAT3, p-EGFR, p-STAT3, DNMT1, and IGFBP2. **G** FCM analysis of CD3^+^CD8^+^IFN-γ cells. **H** The levels of perforin, granzyme A, and B were detected by RT-PCR. **I** The levels of IL-2, IFN-γ, and IFN-α in serum. **J** Perforin, granzyme A, and B levels in the supernatant were tested via ELISA. *P < 0.05 vs. si-NC, #*P* < 0.05 vs. si-DNMT1 + oe-NC

Finally, to further investigate whether inhibiting DNMT1 expression could regulate the IGFBP2/EGFR/STAT3/PD-L1 pathway, RM-1 and DU145 cells were transfected with si-DNMT1 and oe-IGFBP2 plasmids. The decrease in DNMT1 expression and increase in IGFBP2 expression indicated successful transfection, with si-DNMT1#2 showing higher transfection efficiency (Fig. 7A). Subsequent experiments were performed using si-DNMT1#2. It was observed that interfering with DNMT1 reduced cell proliferation, migration, and invasion ability while overexpressing IGFBP2 enhanced these abilities (Fig. 7B and C). Furthermore, interfering with DNMT1 decreased the protein expression of DNMT1, IGFBP2, PD-L1, p-EGFR, and p-STAT3 in cells, while overexpressing IGFBP2 weakened the inhibitory effect of DNMT1 (Fig. 7D and E). Additionally, the COIP results showed that DNMT1 could interact with IGFBP2 protein (Fig. 7F). Based on the above, it was speculated that inhibiting the expression of DNMT1 could suppress the IGFBP2/EGFR/STAT3/PD-L1 pathway, thereby inhibiting the progression of PCa. After co-culturing RM-1 cells with CD8^+^ T cells, it was found that compared to the si-NC group, interference with DNMT1 led to an increase in the positive rate of CD3^+^CD8^+^IFN-γ cells, as well as in the levels of perforin, granzyme A and B in the CD8^+^T cells and cell supernatant. The overexpression of IGFBP2 resulted in a decrease in the levels of these indicators, weakening the inhibitory effect of DNMT1 (Fig. 7G, H, I and J). In conclusion, inhibiting the expression of DNMT1 could activate the cytotoxic effect of CD8^+^ T cells by suppressing the IGFBP2/EGFR/STAT3/PD-L1 pathway.

## Discussion

PCa is a widespread solid organ cancer that poses a high risk of mortality in men [45]. Therefore, there is an urgent need to develop new anti-tumor mechanisms and stable drugs. ICA and CUR have been demonstrated to possess inhibitory effects on the growth of PCa cells [16, 34]. Additionally, our previous studies have also found that combined treatment with ICA and CUR could inhibit PCa development [18]. In this study, the impact of ICA and CUR on the gut microbiota, metabolism, and immunity of PCa were explored. Through a series of studies, it was found that ICA-CUR mediates the inhibition of PCa by regulating immunity and metabolism. This study is also innovative as no prior studies have reported the effects of combining ICA and CUR on the gut microbiota, metabolism, and immunity in PCa.

In recent years, there has been increasing interest in the role of gut microbiota in diseases, with studies reporting a possible association between gut microbiota and PCa [46]. The gut microbiota consists of microorganisms residing in the gastrointestinal system that interact with the host to regulate behavioral and biochemical processes in the gut [47]. There is increasing evidence indicating a connection between gut microbiota and cancer, which has been confirmed in cancers such as liver cancer [48, 49]. Furthermore, the role of the gut microbiota in drug metabolism, especially in drugs that are difficult to absorb orally, is well known. Therefore, a better understanding of the gut microbiota could lead to new ways of understanding drugs [50]. A mouse subcutaneous RM-1 cell tumor model was first established, and then treated with ICA and CUR, and performed 16 S rRNA sequencing. We found that ICA-CUR affected PCa development and altered the gut microbiota and SCFAs. Through FMT, we found that the ICA-CUR-mediated gut microbiota affected inflammation, immunity, and PCa development, suggesting that the ICA-CUR-mediated gut microbiota may play a vital role in PCa.

Research has shown that methyltransferase DNMT1 promotes the development of PCa [51]. Additionally, ICA and CUR could inhibit the expression of DNMT1 [22, 24]. IGFBP2 is a potential therapeutic target for cancer treatment [52]. Currently, there is no research indicating the relationship between DNMT1, IGFBP2, and PCa, and it is also unknown whether ICA and CUR could regulate the expression of DNMT1 and IGFBP2. Our study demonstrates that DNMT1 and IGFBP2 are highly expressed in PCa, and ICA-CUR could suppress the expression of DNMT1 and IGFBP2. Furthermore, ICA-CUR could inhibit tumor growth in mice. Results from in vitro experiments show that ICA-CUR could inhibit the proliferation, migration, and invasion of RM-1 and DU145 cells. Additionally, both in vivo and in vitro experiments show that DNMT1 positively controls the expression of IGFBP2 and its downstream target proteins. Based on these findings, we speculate that ICA-CUR could inhibit the development of PCa by suppressing IGFBP2/DNMT1.

The gut microbiome plays a crucial role in regulating immune function, leading to diverse responses to immune checkpoint therapy [53]. Terrisse et al. also found that the immune system and gut microbiota impacted the androgen deprivation strategy in treating PCa [6]. Peiffer et al. investigated the stool of patients with advanced metastatic castration-resistant PCa and found that the relationship between gastrointestinal microbiota and immunotherapy response may be specific to the cancer type and/or previous treatment history [54]. Cellular cytotoxicity T cells have been reported as major participants in immune responses, and their activation requires the involvement of perforin, granzyme A, and granzyme B [55]. Our research results demonstrate a correlation between gut microbiota and immune-related markers granzyme A and B. Treatment with ICA-CUR could inhibit the cytotoxic effects of CD8^+^ T cells, increase the positivity rate of CD8^+^ T cells, as well as the levels of perforin, granzyme A, granzyme B, and serum IFN-γ and IFN-α. This indicates that ICA-CUR could enhance the killing function of T cells and exert a stronger anti-tumor effect.

SCFAs are metabolites of the microbiome that have a role in regulating immunity and inhibiting histone deacetylases [56]. The production of SCFAs in the gut microbiota is influenced by various host, environmental, dietary, and microbial factors [57]. It has been reported that the gut microbiota regulates systemic and local prostate insulin-like growth factor 1 through the action of SCFAs to promote the proliferation of PCa cells [9]. Furthermore, in a PCa mouse model, Matsushita et al. found that the gut microbiota and their metabolites, SCFAs, promote cancer growth [58]. Consequently, reducing SCFAs levels in the gut by modifying the microbiome structure could be an effective therapeutic measure for cancer prevention/treatment [59]. Our current research report indicates a connection between SCFAs and gut microbiota. After treatment with ICA + CUR, the abundance of *Akkermansia* and *Dubosiella* decreased at the genus level. Correspondingly, the concentrations of acetic acid, butyric acid, and propionic acid also noticeably decreased after ICA + CUR treatment. Research reported that *Akkermansia* are important producers of SCFAs, primarily acetic and butyric acid [40]. Yuan et al. found that allicin could improve intestinal injury by affecting the production of acetic and propionic acid by *Dubosiella* [41]. The decrease in these SCFAs may be caused by the reduction in gut microbiota levels induced by ICA + CUR. Treatment with ICA-CUR has been shown to affect and suppress SCFAs levels. Dietary composition, circulating metabolite levels, and downstream signaling may be modifiable risk factors for fatal PCa [60]. Besides, IGFBPs are important mediators of the influence of nutrition on growth and play key roles in PCa development [61]. Wu et al. reported that the IGF-IR signaling pathway might regulate androgen receptor compartmentation, thereby changing AR activity in PCa cells [62]. Similarly, Li et al. demonstrated that IGFBP2 regulates PD-L1 levels by activating the EGFR-STAT3 pathway [28]. In this study, we found that ICA-CUR also affects the IGFBP2/EGFR/STAT3/PD-L1 pathway. In vivo, experimental results show that SCFAs promote tumor growth in mice. Additionally, SCFAs could reduce the cytotoxic effects of CD8^+^ T cells, as well as the levels of perforin, granzyme A, granzyme B, IFN-γ, and IFN-α in serum. Further experimental results indicate that SCFAs positively regulate IGFBP2, as well as downstream targets PD-L1, p-EGFR/EGFR, and p-STAT3/STAT3. This suggests that ICA-CUR may exert its effects by modulating IGFBP2 through the inhibition of SCFAs, thereby inhibiting the development of PCa.

We further validated our results through in vitro cell experiments. The results showed that DNMT1 interacts with IGFBP2. After inhibiting the expression of DNMT1, cell migration, invasion, and the cytotoxic effects of CD8^+^ T cells were suppressed, and the €levels of IGFBP2 and its downstream proteins were reduced. On the other hand, overexpression of IGFBP2 had the opposite effect compared to DNMT1 inhibition. Taken together, these results indicate that inhibiting DNMT1 could suppress the EGFR/STAT3/PD-L1 pathway by inhibiting IGFBP2, thereby inhibiting the development of PCa. Our research results for the first time demonstrate the regulatory relationship between DNMT1 and IGFBP2. Additionally, the inhibitory effect of ICA-CUR on PCa development is stronger than using ICA or CUR alone. Therefore, it could be concluded that ICA-CUR affects immune regulation and the development of PCa through the DNMT1/IGFBP2 pathway.

In this study, we preliminarily evaluated the impact of ICA-CUR on PCa and determined whether its potential mechanism of action is related to gut microbiota, metabolism, and immunity. The study reported that ICA and CUR did not show significant toxic effects, and patents related to the preparation of ICA-related drugs have been published [63, 64]. Therefore, the combination of ICA and CUR is safe to some extent and these drugs are commonly used in China without imposing a significant economic burden on patients. Additionally, gut microbiota metabolizes the active ingredients of traditional Chinese medicine, which can help maintain intestinal health by balancing the microbial population [37]. ICA-CUR exerted its action by affecting gut microbiota-SCFAs in vivo, rather than being an invasive treatment. ICA-CUR possesses certain therapeutic potential, and our study may provide a new option for the treatment of PCa. Although studies have reported that ICA and CUR do not exhibit significant toxic effects, the safety and potentially toxic side effects of the combination of ICA and CUR still need further investigation. We have preliminarily confirmed that ICA-CUR hinders the development of PCa by affecting the gut microbiota-SCFAs-IGFBP2 axis, but we have not conducted long-term studies on the effects of ICA-CUR on gut microbiota, and the optimal treatment duration with ICA-CUR for PCa still needs further clarification. Although the study has reported an association between gut microbiota and PCa and its subtypes [65], both PCa and gut microbiota are complex, and it is unclear whether PCa can be treated in all its subtypes. Our future research focus will be on the treatment of different subtypes of PCa with ICA-CUR and the variability in treatment outcomes. Furthermore, due to the heterogeneity of patients, the response of patients after ICA-CUR treatment may vary. At this stage, it is not possible to assess the efficacy and potential side effects of ICA-CUR through clinical feedback data. Therefore, we plan to conduct long-term monitoring on PCa mice treated with ICA-CUR in future experiments, including monitoring of mental status, body weight, body temperature, and biochemical markers. This may help us further evaluate the safety of ICA-CUR. This may assist us in applying ICA-CUR for treating PCa in the future.

## Conclusion

In conclusion, we initially explored the effect of ICA-CUR on PCa and determined whether the underlying mechanism was related to the gut microbiota, metabolism, and immunity. Through in vivo and in vitro experiments, the ICA-CUR-mediated gut microbiota and SCFAs inhibited PCa by regulating immunity. In addition, we have also found that ICA-CUR could affect cancer cell development and the level of PD-L1 by targeting the DNMT1/IGFBP2 axis, which in turn influences the differentiation of immune T cells. On the other hand, ICA-CUR could inhibit the gut microbiota-SCFAs, and SCFAs could regulate IGFBP2 levels, thus affecting the EGFR/STAT3/PD-L1 pathway and the differentiation of immune T cells, ultimately inhibiting PCa development. These results indicate an alternative mechanism underlying ICA-CUR and gut microbiota, metabolism, and immunity. This study provides a reference and basis for future clinical treatment of PCa and provides a new strategy for treating PCa.

### Supplementary Information

Supplementary Material 1.

Supplementary Material 2.

Supplementary Material 3.

Supplementary Material 4.

## Data Availability

Data will be made available on request.

## Abbreviations

PCa: Prostate cancer
SCFAs: Short-Chain Fatty Acids
IGFBP2: Insulin-like Growth Factor (IGF) Binding Protein 2
IGFBP3: Insulin-like Growth Factor (IGF) Binding Protein 3
ICA: Icariin
CUR: Curcumol
STAT3: Signal Transducer and Activator of Transcription 3
DNMT1: DNA methyltransferase 1
PD-L1: Programmed Death-Ligand 1
DOC: Docetaxel
FMT: Fecal Microbiota Transplantation
IFN-γ: Interferon-γ
IFN-α: Interferon-γ
