# Supplementary figures and images for: Icaritin-curcumol activates CD8+ T cells through regulation of gut microbiota and the DNMT1/IGFBP2 axis to suppress the development of prostate cancer
Source: J Exp Clin Cancer Res. 2024 May 23;43:149. doi: 10.1186/s13046-024-03063-2 (PMC11112810; doi:10.1186/s13046-024-03063-2)

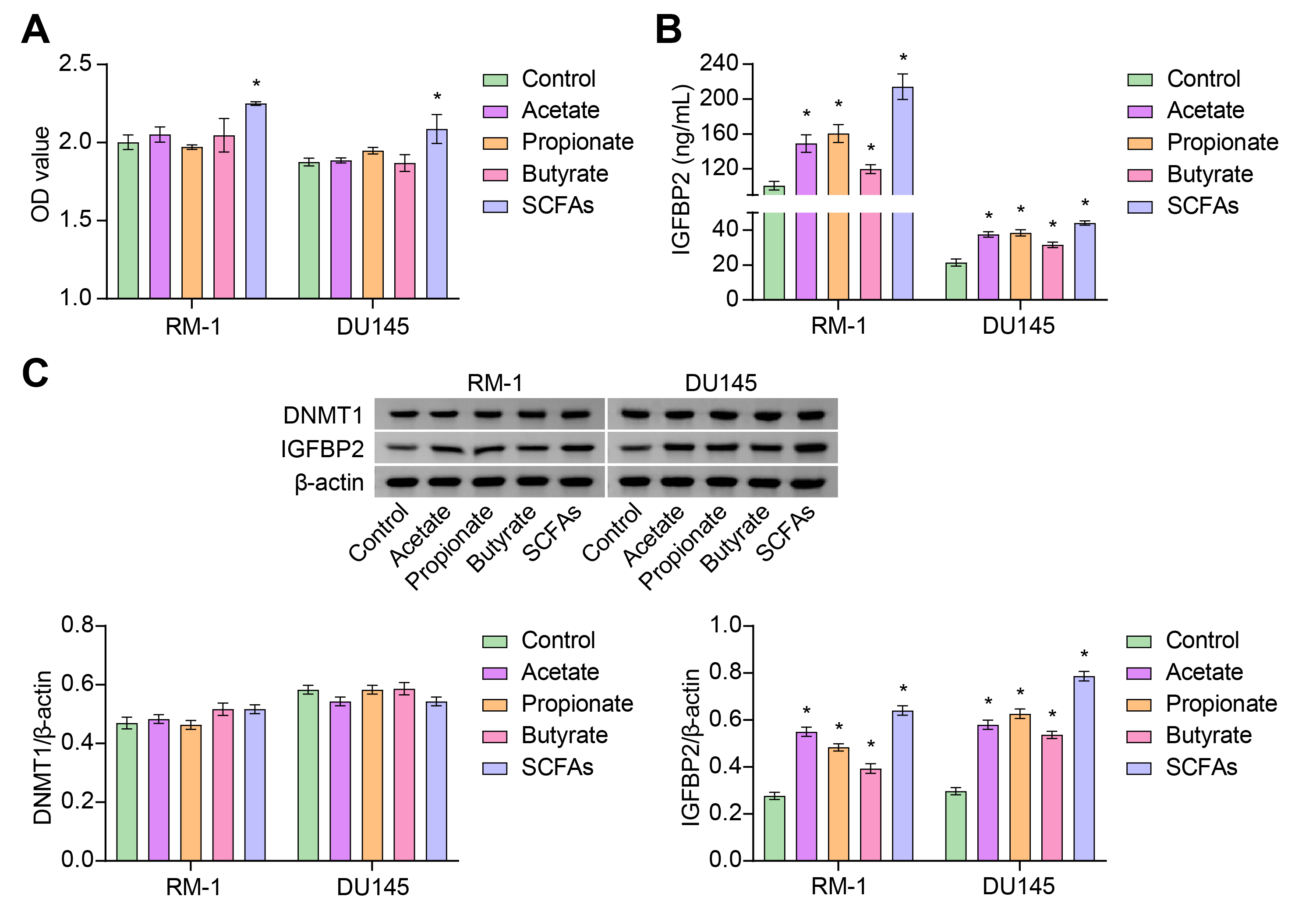

Supplement: Supplementary file 1 — Supplementary Material 1. [file 13046_2024_3063_MOESM1_ESM.jpg]

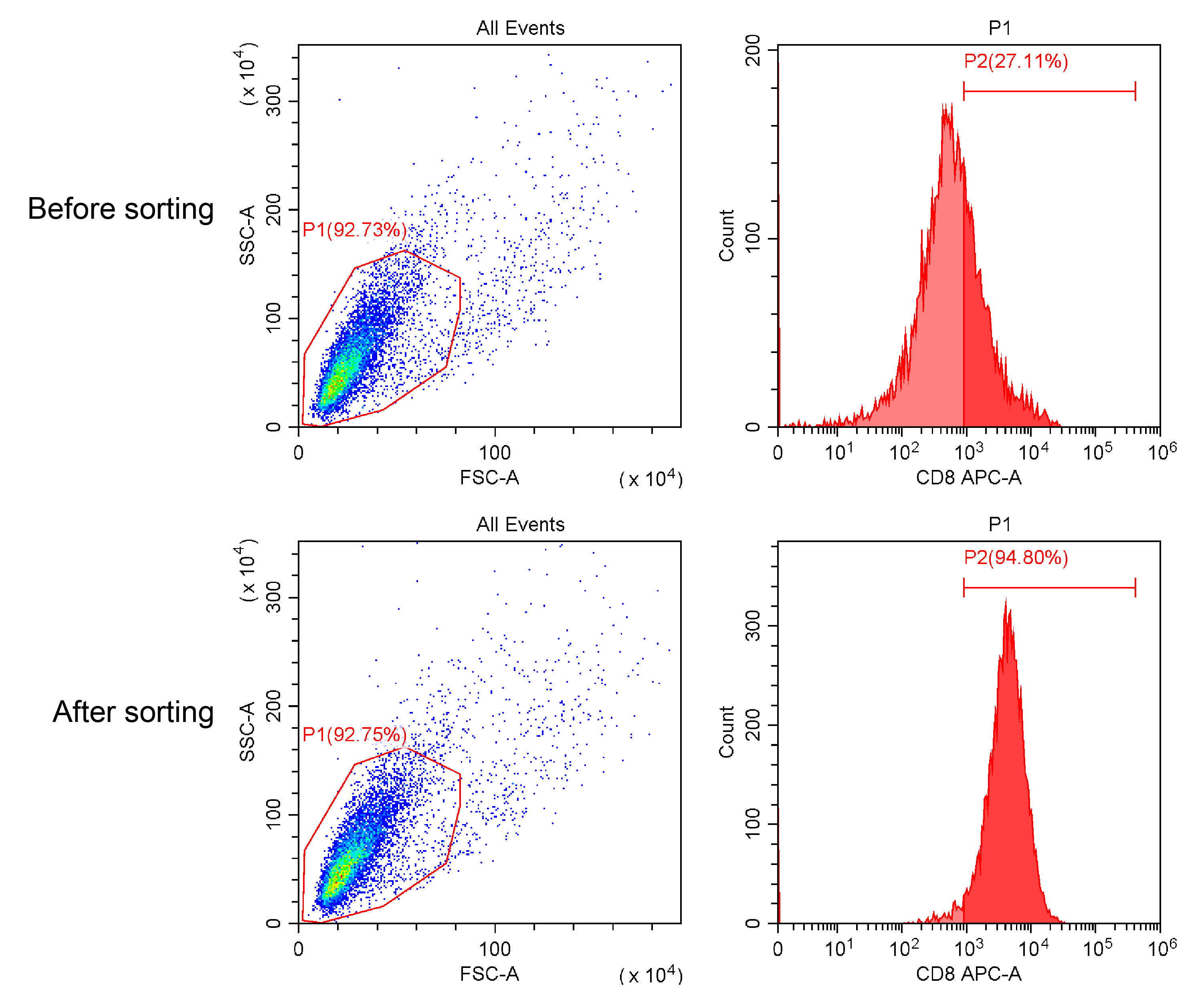

Supplement: Supplementary file 2 — Supplementary Material 2. [file 13046_2024_3063_MOESM2_ESM.jpg]

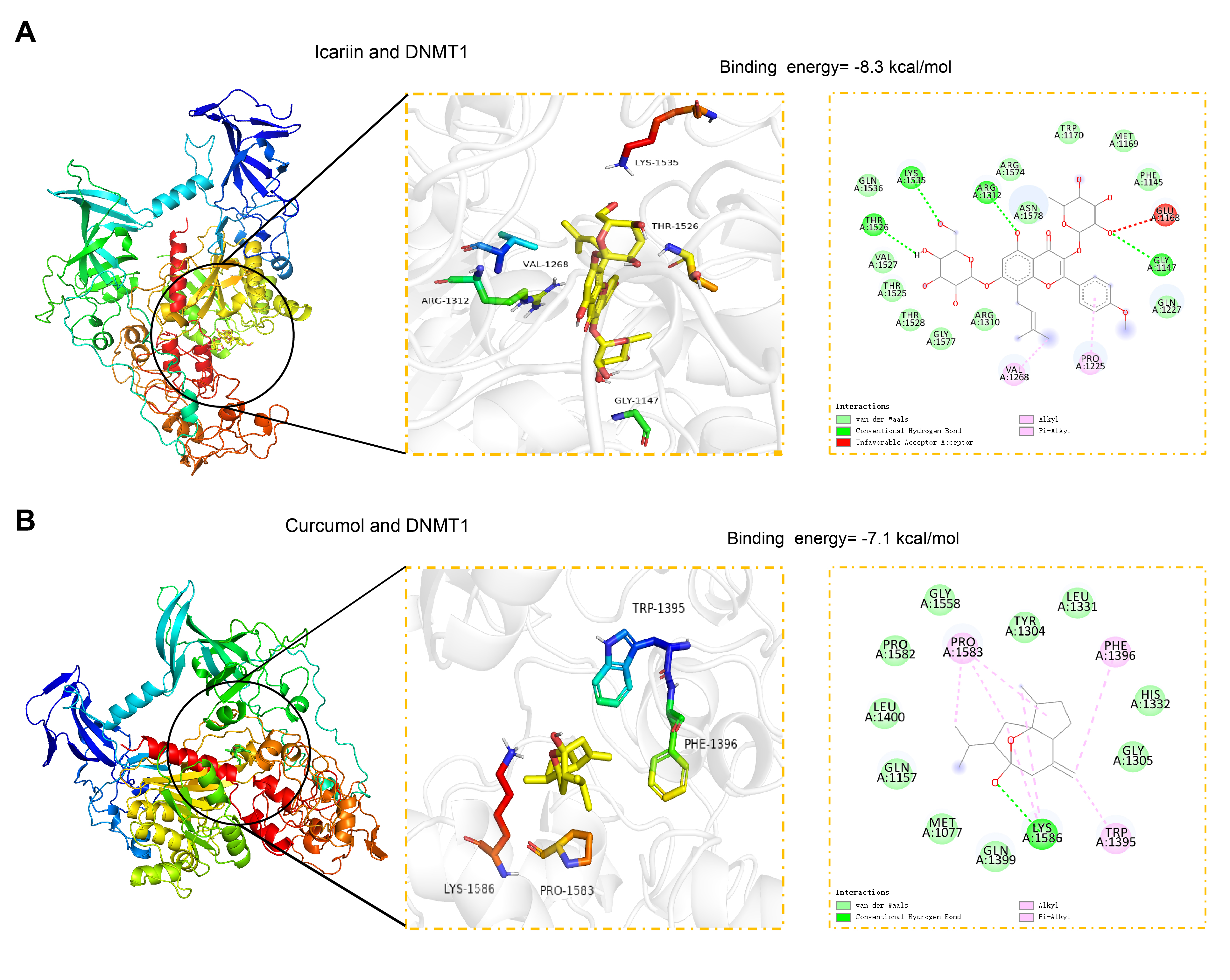

Supplement: Supplementary file 3 — Supplementary Material 3. [file 13046_2024_3063_MOESM3_ESM.jpg]
