# Supplementary material for: Icaritin-curcumol activates CD8+ T cells through regulation of gut microbiota and the DNMT1/IGFBP2 axis to suppress the development of prostate cancer
Source: J Exp Clin Cancer Res. 2024 May 23;43:149. doi: 10.1186/s13046-024-03063-2 (PMC11112810; doi:10.1186/s13046-024-03063-2)
Supplement: Supplementary file 4 — Supplementary Material 4. [file 13046_2024_3063_MOESM4_ESM.docx]

Dear Editor,

Thanks for your work on our manuscript. These comments and suggestions are very valuable and can significantly improve the quality of our manuscript. We have addressed all the issues in this response letter and revised the manuscript. All changes in the manuscript were highlighted in red. We do hope this helps you better understand our work.

**Manuscript title:** Icaritin-Curcumol activates CD8^+^ T cells through regulation of gut microbiota and the DNMT1/IGFBP2 axis to suppress the development of prostate cancer

**Manuscript ID: JECC-D-24-00213**

Best regards,

Wen Sheng

**REFEREE 1**
**Comments:** Prostate cancer (PCa) incidence and mortality rates are rising. The authors have previously shown that the combination of icariin (ICA) and curcumol (CUR) induced autophagy and ferroptosis in PCa cells and altered lipid metabolism. Now they aimed to further explore the effects of the combination of ICA and CUR on gut microbiota, metabolism, and immunity in PCa. Using various *in vivo* and *in vitro* models, the authors demonstrated that after treatment with ICA-CUR, there was a decrease in tumor volume and weight, accompanied by changes in gut microbiota. ICA-CUR affected SCFAs and DNMT1/IGFBP2/EGFR/STAT3/PD-L1 pathway. ICA-CUR increased the positive rates of CD3^+^CD8^+^IFN-γ, CD3^+^CD8^+^Ki67 cells, and the levels of IFN-γ and IFN-α in the serum. A combinatorial treatment with ICA-CUR and FMT decreased tumor volume and weight. SCFA promotes tumor development and the expression of DNMT1, and IGFBP2. *In vitro*, DNMT1/IGFBP2 promotes cell migration and proliferation. ICACUR inhibited the expression of DNMT1/IGFBP2. In conclusion, ICA-CUR treatment mediates the interaction between gut microbiota and the DNMT1/IGFBP2 axis to inhibit the progression of prostate cancer by regulating immune response and metabolism, suggesting a potential therapeutic strategy for PCa.

The results are interesting and potentially important for prostate cancer treatment. However, there are several issues to modify/clarify for improving the manuscript:

Reply: Thank you for your affirmation of our research and experimental results. We agree that there are areas in the manuscript that need further modification or clarification, and will address these problems to improve the overall quality of the manuscript.

Referee 1/problem 1: While it is clear that the treatment changes the composition of the microbiota, it is not very clear how this change affects/connects to the DNMT1/IGFBP2 pathways. The evidence of this link is not really supported as the data are presented. Thus, the microbiota component remains purely correlative and does not add much to the whole story. The paper has already a lot of data and the Microbiota data can be eliminated without detracting from the full picture of the data presented.

Reply: Thank you for reviewing our manuscript and providing your valuable comments. Your suggestions are very constructive and have been greatly helpful in improving the quality of our manuscript. As you mentioned, the treatment with icaritin-curcumol (ICA-CUR) significantly alters the composition of gut microbiota, but how this change affects/connects to the DNMT1/IGFBP2 pathways is still unclear. Our current data also did not fully explain the rationale of this pathway. As a result, the composition of the gut microbiota appeared relatively isolated and not well linked with subsequent experiments. However, after double-checking the content of the manuscript, we realized that the description of the results of the changes in the gut microbiota at the genus level after ICA-CUR treatment was omitted from the original manuscript. We have adjusted and added content to this section. Specifically, there was a significant decrease in *Akkermansia* and *Dubosiella* in the mouse intestine after ICA-CUR treatment. *Akkermansia* and *Dubosiella* have been reported to be associated with short-chain fatty acids (SCFAs) [1, 2]. Our results demonstrated that SCFAs (acetic acid, butyric acid, and propionic acid) decreased after ICA-CUR treatment. Consequently, we speculated that ICA-CUR could lower acetic acid, butyric acid, and propionic acid by downregulating the abundance of *Akkermansia* and *Dubosiella*. Including this section enhances the overall coherence of the manuscript. In addition, to ensure coherence throughout the entire article, we carried out additional *in vitro* cell experiments using salts of acetate, propionate, and butyrate, as well as a combination of the three SCFAs (cells were treated with acetate, propionate, and butyrate simultaneously), to investigate their impact on Prostate cancer (PCa). We hope that after reviewing our additional results and descriptions, your concerns will be addressed. The specific content is as follows:

The literature reports that gut microbiota plays a key role in the metabolism of SCFAs and can influence the expression of SCFAs [3, 4]. Gut microbiota, through the action of SCFAs, regulate host systemic and local prostate IGF1, promoting PCa cell proliferation [5]. Our study confirmed that *Akkermansia* and *Dubosiella* at the genus level in the intestines of mice significantly decreased after treatment with ICA-CUR, altering the composition of gut microbiota (Figure 1G). Additionally, acetic acid, butyric acid, and propionic acid also significantly decreased after ICA-CUR treatment (Figure 1I). *Akkermansia* is an important SCFAs-producing bacterium, mainly producing acetic acid and butyric acid [1]. Yuan *et al.* found that allicin can improve intestinal damage by affecting the production of acetic acid and propionic acid by *Dubosiella* [2]. Therefore, the reduction in acetic acid, butyric acid, and propionic acid may be due to the downregulation of *Akkermansia* and *Dubosiella* by ICA-CUR. In addition, we made a fecal microbiota suspension from ICA-CUR treated feces and administered it to mice by gavage needle, and found that tumor growth was inhibited. These results indicated that ICA-CUR affected Pca through gut microbiota. Lastly, we conducted additional *in vitro* cell experiments with acetate, propionate, and butyrate salts as well as SCFAs, and found that compared to the Control group, there was no significant change in cell viability for the Acetate, Propionate, and Butyrate groups of RM-1 and DU145 cells, but the cell viability increased in the SCFAs group. It was proved that SCFAs promoted the proliferation of PCa cells *in vitro*. Considering our previous results, ICA-CUR reduces the enrichment of SCFAs by affecting gut microbiota, especially *Akkermansia* and *Dubosiella*, thereby mediating the development of PCa.

Furthermore, after careful consideration, we do not delete the microbiota data for the following reasons reason: increasing evidence suggests a connection between gut microbiota and cancer, which has been confirmed in cancers such as liver cancer [6, 7]. Liu *et al.* reported that ICA is easily hydrolyzed by intestinal bacteria or enzymes after oral administration [8]. CUR may improve liver inflammation by influencing the gut microbiota [9]. It has been reported that SCFAs can regulate host systemic and local prostate insulin-like growth factor 1 (IGF1), promoting PCa cell proliferation [5]. Additionally, IGFBP2 is an important oncogene in various human cancers [10]. It is speculated that gut microbiota-SCFAs may affect the progression of PCa by regulating insulin-like growth factor binding protein 2 (IGFBP2) levels. Our experimental results in section 3.3 demonstrate that after the mice were treated with fecal microbiota transplantation (FMT) with donors from the PCa and ICA-CUR, IGFBP2 and its downstream target proteins in mice were inhibited. Hence, we have demonstrated that gut microbiota-SCFAs may affect PCa by influencing IGFBP2. Deleting gut microbiota data would result in insufficient research content that cannot support our experimental design or explain our subsequent experiments. Lastly, considering the richness of the results currently presented, and our further experiments involving FMT to elucidate the effect and mechanisms of gut microbiota in PCa, previous correlation analyses related to gut microbiota appear somewhat redundant and do not establish a clear link or explanation between the contents of the article. Therefore, we have removed this section of results to streamline the manuscript.

The additional experiments we have included are as follows (Materials and Methods, lines 233-238, Results 3.1, lines 372-414;3.6, lines 537-565):

Materials and Methods

In experiment 5, RM-1 and DU145 cells were grouped into the Control, Acetate, Propionate, Butyrate, and SCFAs groups. In the Control group, cells were cultured normally. In the Acetate group, cells were treated with acetate for 72 h. In the Propionate group, cells were treated with propionate for 72 h. In the Butyrate group, cells were treated with butyrate for 72 h. In the SCFAs group, cells were treated with acetate, propionate, and butyrate together for 72 h.

Results

Figure 1. ICA-CUR inhibits the development of PCa and regulates gut microbiota and SCFAs. A. Tumor imaging, volume, and weight measurements. B. IF staining to examine changes in Ki67 expression in tumors. C. Venn diagram analysis of differential OUTs among the different groups. D. Principal component analysis plot. E. α-diversity analysis (Observed OTUs, Simpson's index, Shannon index, Chao1 index, PD whole tree). F. β-diversity analysis. G. Changes in the relative abundance of gut microbiota at the phylum and genus levels. H. LefSe analysis of overall microbial structure differences at the phylum and genus levels. I. Changes in SCFAs (acetic acid, propionic acid, isobutyric acid, butyric acid, isovaleric acid, and valeric acid) in mouse feces. *P<0.05 vs PCa.

3.6 ICA-CUR inhibits the development of PCa, the DNMT1/IGFBP2 pathway, and activates cytotoxic effects of CD8^+^ T cells *in vitro*

In previous experiments, it has been demonstrated that ICA+CUR may mediate the development of PCa and T cell immunity through the gut microbiota-SCFAs-IGFBP2 pathway. *In vivo* experiments have shown that ICA-CUR inhibits the production of SCFAs. However, considering the relatively low overall content of isobutyric acid and isovaleric acid, the effects of acetate, propionate, butyrate, and SCFAs on PCa were first investigated *in vitro.* Compared to the Control group, there was no significant change in the cell viability of RM-1 and DU145 cells in the acetate, propionate, and butyrate groups, but the cell viability in the SCFAs group increased, indicating that SCFAs promote the proliferation of RM-1 and DU145 cells (Figure S1A). Compared to the Control group, the levels of IGFBP2 in RM-1 and DU145 cells were significantly increased in the acetate, propionate, butyrate, and SCFAs groups, while DNMT1 showed no significant change (Figure S1B-1C). These results further confirm that SCFAs could directly promote the level of IGFBP2 and that SCFAs promote the progression of PCa.

Figure S1, Cells were treated with different concentrations of acetate, propionate, and butyrate salts, and then cell viability was measured, along with changes in IGFBP2 and DNMT1 levels within the cells.

Referee 1/problem 2: The Result section can be improved. The results of the Experiments and their interpretation can be improved as the correlation with the figure panels.

Reply: Thanks for kindly reminding us, and your suggestions have helped improve the quality of our manuscript. In light of your suggestions, we have recognized the necessity for enhancing the description of the experimental results. Consequently, we have conscientiously revised the Results section based on your suggestions, strengthening the connection between the Figures and Results descriptions. We aim to elucidate our experiment more effectively for reader comprehension.

For example, the revisions are shown below (Results 3.1):

3.1. ICA-CUR inhibits the development of PCa and regulates gut microbiota and SCFAs

To investigate the effect of combined ICA and CUR treatment on PCa, a mouse subcutaneous RM-1 cell tumor model was first established. Subsequently, the mice were respectively treated with ICA, CUR, ICA-CUR, and DOC (positive control), and changes in tumor size were observed. As compared to the PCa group, tumor size and weight witnessed a decrease in the ICA, CUR, ICA+CUR, and DOC groups, with the most substantial reduction observed in the ICA+CUR group (Figure 1A). Ki67 is a marker protein associated with cancer cell proliferation, expressed in various cancers, and used as a prognostic marker [11, 12]. Moreover, Ki67 expression levels were noted to be lower in the ICA, CUR, ICA+CUR, and DOC groups than those in the PCa group. Noticeably, the expression of Ki67 in the ICA+CUR group witnessed the most significant decline (Figure 1B). The above results preliminarily indicated that the efficacy of ICA combined with CUR in treating PCa was significantly better than when ICA and CUR were used alone. The gut microbiota metabolizes the active ingredients of traditional Chinese medicine, which can help maintain intestinal health by balancing the microbial population [13]. Researchers have found that ICA is easily metabolized by gut microbiota or enzymes after oral administration [8]. Additionally, CUR may improve liver inflammation by influencing the gut microbiota [9]. Therefore, the changes in gut microbiota following treatment with ICA+CUR were further analyzed using 16S rRNA sequencing. Figure 1C showed differences in OTU among groups. Compared to the PCa group, the number of gut microbiota in the ICA and ICA+CUR groups increased. Principal component analysis showed the degree of dispersion in each sample group. Compared to the PCa group, the microbial communities of the ICA and ICA+CUR group samples were significantly different (Figure 1D). Besides, the alpha diversity (Observed, Chao1, ACE, Shannon, Simpson index) and β-diversity results of the mice showed that the gut microbiota in the ICA+CUR group was the most abundant, and there was a significant difference between the ICA+CUR group and the PCa group, indicating that the treatment of the ICA+CUR group may have an impact on the gut microbiota (Figure 1E-1F). Further investigation revealed changes in the gut microbiota of mice at the phylum and genus levels, as well as the overall structural differences at the phylum and genus levels. It was observed that after ICA+CUR treatment, the levels of Akkermansia, and Dubosiella within the gut of the mice significantly decreased at the genus level (Figures 1G-1H). Research reported that Akkermansia are important producers of SCFAs, primarily acetic and butyric acid [1]. Yuan *et al.* found that allicin could improve intestinal injury by affecting the production of acetic and propionic acid by Dubosiella [2]. In addition, the changes in SCFAs in the mouse feces showed a significant decrease in acetic acid, butyric acid, and propionic acid after ICA+CUR treatment (Figure 1I). This result corresponded to the decrease at the genus level of Akkermansia, and Dubosiella after ICA+CUR treatment. Based on the above results, it was indicated that ICA+CUR inhibits the development of PCa and could regulate gut microbiota and SCFAs changes. Additionally, ICA+CUR may suppress the levels of SCFAs by inhibiting gut microbiota.

Figure 1. ICA-CUR inhibits the development of PCa and regulates gut microbiota and SCFAs. A. Tumor imaging, volume, and weight measurements. B. IF staining to examine changes in Ki67 expression in tumors. C. Venn diagram analysis of differential OUTs among the different groups. D. Principal component analysis plot. E. α-diversity analysis (Observed OTUs, Simpson's index, Shannon index, Chao1 index, PD whole tree). F. β-diversity analysis. G. Changes in the relative abundance of gut microbiota at the phylum and genus levels. H. LefSe analysis of overall microbial structure differences at the phylum and genus levels. I. Changes in SCFAs (acetic acid, propionic acid, isobutyric acid, butyric acid, isovaleric acid, and valeric acid) in mouse feces. *P<0.05 vs PCa.

Referee 1/problem 3: The paper is full of abbreviations and sometime difficult to read.

Reply: Thanks for reminding us and we are grateful for the question you pointed out. For the abbreviations mentioned in your query, we have provided the full names at their first mention in the text. After carefully considering your suggestion, we have appropriately removed some abbreviations and replaced them with their full names to reduce confusion for readers of this paper, such as insulin-like growth factor 1 (IGF1). Nevertheless, for terms like Icaritin-Curcumol (ICA) and short-chain fatty acids (SCFAs), using abbreviations in the text makes the article more concise and facilitates both title writing and mechanism organization. Additionally, we have compiled key abbreviations in the order they appeared in this article, and included them at the end of the manuscript. We hope that the Abbreviations will assist you in reading the manuscript.

Abbreviations

PCa: prostate cancer;

SCFAs: short-chain fatty acids;

IGFBP2: insulin-like growth factor binding protein 2;

IGFBP3: insulin-like growth factor binding protein 3

ICA: icariin;

CUR: curcumol;

STAT3: signal transducer and activator of transcription 3

DNMT1: DNA methyltransferase 1;

PD-L1: programmed death-ligand 1;

DOC: docetaxel;

FMT: fecal microbiota transplantation;

IFN-γ: interferon-γ;

IFN-α: interferon-γ;

Referee 1/problem 4: In the Introduction, referring to prostate cancer: "It is a complex and heterogeneous disease with a variable natural history" What do the authors mean? Not clear.

Reply: Thank you very much for your careful review, we have revised the section for better understanding. In this section, we aim to describe prostate cancer and its causes, laying the groundwork for the introduction of our research later on. We have made changes to the description of this part. The updated section is as follows:

The revisions are shown below (Introduction, lines 55-58):

Prostate cancer (PCa) is a malignant tumor affecting men and is an important cause of the global increase in male mortality [14]. The causes of PCa involve multiple factors [15]. Even with second-generation androgen receptor axis inhibitors as the primary treatment option, most metastatic cases progress to castration-resistant PCa after the initial treatment response with a poor prognostic outcome [16]. Therefore, new treatments are required to enhance the prospects for PCa patients.

Referee 1/problem 5: In the Methodology section the authors report: "moisten the cell screen" What does it mean? Not clear.

Reply: Many thanks for your kind reminder. Based on your feedback, we have reviewed the article and found two instances of mentioning "moisten the cell screen," located in sections 2.3 and 2.10 respectively. The intended meaning in both cases is to “moisten the cell strainer with a cell culture medium”. Here our wording is not precise enough, so we have revised the wording in these sections to ensure a smoother reading experience for the readers.

The revisions are shown below (Materials and Methods, 2.3, lines 176-177, 2.10, lines 285-286):

2.3. Magnetic bead sorting of CD3^+^CD8^+^ T cells

A complete culture medium (2 mL) was taken to moisten a 40 μm cell strainer before placing the mouse spleen. The mouse spleen was cut with ophthalmic scissors and ground by pressing the bottom of the piston of a 5 mL syringe. The strainer was rinsed with 10 mL of RPMI-1640 complete medium (C3010-0500, Viva Cell). Cell precipitation was achieved by centrifugation at 1500 rpm.

2.10. Flow cytometry (FCM)

A complete culture medium (2 mL) was taken to moisten a 40 μm cell strainer before placing the tumor. The tumor was cut with ophthalmic scissors and ground by pressing the bottom of the piston of a 5 mL syringe. The rest of the operation is as described above for magnetic bead sorting. RM-1 and DU145 cells were resuspended in 500 μL of 10% FBS 1640, added 1μL of Cell Stimulation Cocktail (plus protein transport inhibitors), and incubated at 37℃. The cells were resuspended in 500 μL 10% FBS 1640, and then 1uL of Cell Stimulation Cocktail (plus protein transport inhibitors) was added. The cells were stimulated at 37℃.

**REFEREE 2**
**Comments:** **The study provides a clear overview of the study's objective and the significance of investigating ICA-CUR in PCa. The study found that ICA-CUR mediates the interaction between gut microbiota and the DNMT1/IGFBP2 axis to inhibit the progression of prostate cancer by regulating immune response and metabolism, suggesting a potential therapeutic strategy for PCa.**

Reply: Thank you very much for affirming our research. Your comments have significantly boosted our confidence. We hope that our study provides a little assistance for the future treatment of PCa.

Referee 2/problem 1: Additional experimental studies investigating the specific mechanisms through which ICA-CUR affects the gut microbiota, such as metagenomic analysis or functional assays, would provide a more comprehensive understanding of the interplay between ICA-CUR and the gut microbiota in the context of prostate cancer.

Reply: Thank you for reviewing our manuscripts and giving us positive comments. We greatly appreciate your valuable comments, which further enhance the quality of our work.

In recent years, there has been increasing interest in the role of gut microbiota in many diseases, with studies reporting a possible association between gut microbiota and PCa [17]. Moreover, gut microbiota metabolizes the active ingredients of traditional Chinese medicine, which can help maintain intestinal health by balancing the microbial population [13]. Researchers have found that H. attenuatum Choisy can regulate the gut microbiota to suppress intestinal inflammation [18]. ICA is easily metabolized by gut microbiota or enzymes after oral administration [8]. Additionally, CUR may improve liver inflammation by influencing the gut microbiota [9]. The gut microbiota plays a crucial role in the metabolism of SCFAs and influences the expression of SCFAs [3, 4]. Our study focuses on exploring the mechanism of action of ICA-CUR in the context of PCa, highlighting the overall impact of ICA-CUR on the gut microbiota and its metabolic product SCFAs. Following careful consideration, we designed comprehensive experiments that provided convincing evidence of how ICA+CUR affects the gut microbiota in mice (Figure 1). Furthermore, research has shown that one of the primary mechanisms by which the gut microbiota regulates anti-tumor immunity is through its metabolites [19]. Metabolites, as small molecules, can diffuse from their initial location in the gut and influence local and systemic anti-tumor immune responses [19]. SCFAs are the main metabolites produced by gut microbiota fermentation [20], and they can regulate host systemic and local prostate IGF1, promoting PCa cell proliferation [5]. IGFBP2 is an important oncogene in various human cancers [10]. We analyzed changes in gut microbiota, SCFAs, and mouse tumors after ICA-CUR treatment, and found that FMT (with donors from the PCa and ICA-CUR group) hindered PCa progression. Hence, upon observing from the aforementioned experimental outcomes that ICA-CUR could suppress gut microbiota and SCFAs, we speculated that the gut microbiota might play a role in mediating the SCFAs-IGFBP2 axis, influencing the development of PCa, which prompted us to delve into further mechanistic investigations. Additionally, we supplemented our *in vitro* analysis of SCFAs' impact on PCa to further validate our *in vivo* experimental results. After treating mice with ICA-CUR, we added SCFAs to their drinking water and observed an increase in the levels of IGFBP2 in the mice, as well as an increase in tumor volume. The levels of perforin, granzyme A, and granzyme B in CD8^+^ T cells, as well as the levels of IFN-γ and IFN-α in the serum, were decreased, indicating that the cytotoxic effects of CD8^+^ T cells were activated. Subsequently, upon treatment with anti-IGFBP2 on the basis of ICA+CUR+SCFAs, the expression of PD-L1, p-EGFR, and p-STAT3 decreased, and tumor volume reduced. Additionally, the levels of perforin, granzyme A, and granzyme B in CD8^+^ T cells, as well as the levels of IFN-γ and IFN-α in the serum, increased, leading to a reversal of the promotive effects of SCFAs. Furthermore, by supplementing SCFAs and assessing their impact on PCa *in vitro*, we observed that SCFAs promoted the proliferation of PCa cells, further confirming our in vivo experimental results. In conclusion, our study demonstrated that ICA-CUR mediated CD8^+^ T cell activation hinders PCa progression by modulating the gut microbiota-SCFAs-IGFBP2 and DNMT1/IGFBP2 axes. Further, inhibition of IGFBP2 activates CD8^+^ T cytotoxic effects by inhibiting the EGFR/STAT3/PD-L1 pathway. Our study provides a potential therapeutic strategy for the treatment of PCa. However, we did not conduct detailed genomic or functional analyses of how ICA-CUR affects the gut microbiota, which is a limitation of our study. In future studies, we plan to delve deeper into the specific mechanisms of the gut microbiota through experiments such as metagenomic analysis or functional assays as you suggested.

Referee 2/problem 2: It would be beneficial to include additional *in vivo* experiments to further elucidate the impact of ICA-CUR on immune response and metabolism, potentially exploring the signaling pathways associated with the observed changes in T-cell immunity and metabolic regulation.

Reply: Thank you for your valuable suggestions on our manuscript. We greatly appreciate your insightful recommendations for further elucidating the *in vivo* effects of ICA-CUR on immune response and metabolism. PD-L1 is an inhibitory receptor expressed by T cells that can suppress the activation of immune T cells [21, 22]. IGFBP2 can regulate PD-L1 expression by activating the EGFR-STAT3 signaling pathway [23]. Our research findings indicate that ICA-CUR could inhibit the progression of PCa *in vivo* by suppressing the IGFBP2/EGFR/STAT3/PD-L1 axis, and activate the cytotoxic effect of immune T cells. Additionally, ICA-CUR could downregulate gut microbiota (*Akkermansia* and *Dubosiella*), consequently impacting SCFAs (acetic acid, butyric acid, and propionic acid) levels, leading to their downregulation. ICA-CUR inhibited the levels of IGFBP2 and its downstream target proteins, while SCFAs have a promoting effect on these indicators. Additionally, SCFAs can regulate host systemic and local prostate insulin-like growth factor 1, promoting PCa cell proliferation [5]. Therefore, we speculated that ICA-CUR could inhibit gut microbiota-SCFAs *in vivo* and that SCFAs could regulate the expression of IGFBP2, thereby influencing the EGFR/STAT3/PD-L1 pathway to modulate immune T cells. We conducted a range of experiments, including T-cell immunity and SCFAs metabolism. Studies have reported that CD8^+^ T cells release perforin, granzyme A, and granzyme B when exerting their cytotoxic effects [24]. Interferon-α and interferon-β are effector factors that enhance CD8^+^ T cell responses [25, 26]. Our study began by isolating CD8^+^ T cells using magnetic bead sorting. Subsequently, it was observed that treatment with ICA-CUR led to an increase in serum levels of IFN-α and IFN-β, as well as elevated levels of perforin, granzyme A, and granzyme B in the tumor, indicating that ICA-CUR activated the cytotoxic effect of CD8^+^ T cells. Additionally, ICA-CUR upregulated the levels of IGFBP2 and its target protein PD-L1, as well as p-EGFR and p-STAT3. Therefore, we found that SCFAs could regulate the levels of IGFBP2, thereby influencing the EGFR/STAT3/PD-L1 pathway to modulate immune T cells. Furthermore, we conducted supplementary *in vitro* experiments to investigate the impact of SCFAs on PCa cells. In conclusion, our study found that ICA-CUR mediated CD8^+^ T cell activation to inhibit prostate cancer progression by modulating the gut microbiota-SCFAs-IGFBP2 and DNMT1/IGFBP2 axes. Further, inhibition of IGFBP2 activates CD8^+^ T cytotoxic effects by inhibiting the EGFR/STAT3/PD-L1 pathway. Our study provides a potential therapeutic strategy for the treatment of PCa. We did not explore other signaling pathways associated with changes in ICA-CUR regulation of T cell immunity and metabolism, which is a limitation of our study. We plan to screen the relevant signaling pathways by experiments such as metagenomic analysis or functional assays in future studies. Finally, we will confirm the specific mechanism of ICA-CUR regulating immunity and metabolism through other pathways by *in vivo* and *in vivo* experiments.

Referee 2/problem 3: Considering the complexity of the gut microbiota and its potential role in mediating therapeutic effects, conducting follow-up experiments to explore the long-term effects of ICA-CUR on gut microbiota composition and functionality would enhance the study's findings.

Reply: Thank you for your careful review of our manuscript. Your suggestion to conduct experiments on the long-term effects of ICA-CUR on the composition and function of gut microbiota could provide stronger support for our current experimental results. We find this suggestion to be highly constructive. Our current study focuses on investigating the role and mechanism of ICA-CUR in the context of PCa, without deeply exploring the long-term effects of ICA-CUR on gut microbiota composition and function. Our current experimental results demonstrate that ICA-CUR could hinder the development of PCa by influencing gut microbiota-SCFAs. Specifically, after treatment with ICA-CUR, the levels of *Akkermansia* and *Dubosiella* in the mice intestines significantly decreased at the genus level, and there was a marked reduction in acetic acid, butyric acid, and propionic acid. ICA-CUR may regulate SCFAs through gut microbiota. Additionally, we considered the complexity of gut microbiota when designing the experiments, making efforts to minimize the influence of experimental conditions on research conclusions and carrying out a rich array of experiments to the best of our ability. However, our current research funding is insufficient to support long-term experiments. We will enhance this aspect in future research endeavors.

Referee 2/problem 4: While the study effectively demonstrates the inhibitory effects of ICA-CUR on tumor progression, incorporating additional analyses to assess potential adverse effects or off-target impacts of the treatment regimen would provide a more balanced evaluation of its overall efficacy and safety profile.

Reply: Thank you for reviewing our manuscripts and giving us positive comments. We greatly appreciate your valuable comments, which further enhance the quality of our work. Your suggestion to incorporate more analysis to evaluate the potential adverse reactions of ICA-CUR is considered very valuable. However, our current research focuses on investigating the effects and mechanisms of ICA-CUR on PCa and has utilized both *in* *vitro* and *in vivo* experiments to demonstrate the inhibitory effects of ICA-CUR on the progression of PCa. Patents have been granted for the use of ICA in preparing immune-modulating drugs for tumors, with findings indicating low toxicity of ICA to normal cells and no apparent adverse reactions [27]. Furthermore, studies by Cai *et al.* have shown that using CUR alone or in combination with celecoxib does not cause liver toxicity and does not lead to significant changes in mouse body weight [28]. These studies suggest that ICA and CUR are not toxic when administered individually. Therefore, our experiments did not further investigate the potential adverse reactions of combined ICA and CUR use, which is also a limitation of our study. We will conduct a more comprehensive evaluation of the overall efficacy and safety of ICA-CUR in future experiments, aiming to provide some support for the treatment of PCa.

Referee 2/problem 5: Given the multifaceted nature of prostate cancer development and progression, addressing potential limitations related to the generalizability of the findings, such as variations in PCa subtypes or stages, would strengthen the translational relevance of the study outcomes.

Reply: Thank you for your detailed review of our manuscript. Hu *et al.* found that ICA can inhibit the development of PCa in TRAMP mice and further confirmed the inhibitory effect of ICA in human LNCaP and PC-3 cells [29]. Wen *et al.* verified the inhibitory effect of CUR on PCa through *in vivo* experiments and *in vitro* cultures of PCa cell lines (PC3, DU145, LNCaP) and normal prostate cells (RWPE-1) [30]. Our study explored the effects of ICA-CUR on RM-1 tumor-bearing mice *in vivo* and further elucidated the effects and mechanisms of ICA-CUR through RM-1 and DU145 cells *in vitro*. RM-1 cells (mouse PCa cells) and DU145 cells (human PCa cells) are widely used in PCa research. Additionally, patents have been granted for the use of ICA in preparing immune-modulating drugs for tumors [27], highlighting the therapeutic potential of combined ICA and CUR in treating PCa. Furthermore, research has indicated an association between gut microbiota and PCa and its subtypes [31], suggesting that modulating gut microbiota may be a potential therapeutic approach for preventing PCa. Our experiments have demonstrated that ICA-CUR influences gut microbiota and their metabolic products, SCFAs. However, given the complexity of PCa and the differences in prognosis and treatment methods among its subtypes, our current research funding does not support investigations into all PCa subtypes. In future experiments, we will further explore this area.

Referee 2/problem 6: The study might benefit from discussing the potential challenges or limitations associated with translating the observed effects of ICA-CUR on gut microbiota, metabolism, and immunity into clinical applications, including considerations of patient heterogeneity and treatment variability.

Reply: Thank you for reviewing our manuscript. Your comments will greatly help improve the quality of our manuscript. Your suggestion regarding the potential challenges or limitations associated with translating the observed effects of ICA-CUR on gut microbiota, metabolism, and immunity into clinical applications is very constructive and will greatly inspire our future research. Although studies have reported that ICA and CUR do not exhibit significant toxic effects, and patents related to the preparation of ICA-related drugs have been published [27, 28], the safety and potentially toxic side effects of the combination of ICA and CUR still need further investigation. We have preliminarily confirmed that ICA-CUR hinders the development of PCa by affecting the gut microbiota-SCFAs-IGFBP2 axis, but we have not conducted long-term studies on the effects of ICA-CUR on gut microbiota, and the optimal treatment duration with ICA-CUR for PCa still needs further clarification. Although the study has reported an association between gut microbiota and PCa and its subtypes [31], both PCa and gut microbiota are complex, and it is unclear whether PCa can be treated in all its subtypes. Our future research focus will be on the treatment of different subtypes of PCa with ICA-CUR and the variability in treatment outcomes. Furthermore, due to the heterogeneity of patients, the response of patients after ICA-CUR treatment may vary. At this stage, it is not possible to assess the efficacy and potential side effects of ICA-CUR through clinical feedback data. Therefore, we plan to conduct long-term monitoring on PCa mice treated with ICA-CUR in future experiments, including monitoring of mental status, body weight, body temperature, and biochemical markers. This may help us further evaluate the safety of ICA-CUR. This may assist us in applying ICA-CUR for treating PCa in the future.

Referee 2/problem 7: Furthermore, providing a more detailed discussion of the potential scalability and feasibility of leveraging ICA-CUR as a therapeutic strategy for prostate cancer treatment could offer valuable insights into the practical applicability of the research findings.

Reply: Thank you for reviewing our manuscript. A more detailed discussion of the potential scalability and feasibility of the complemented ICA-CUR, as a therapeutic strategy for PCa, which you have proposed, is valuable and warranted. We have demonstrated that ICA-CUR is more effective in the treatment of PCa than either treatment separately. Moreover, we mentioned in the previous question that ICA and CUR did not show significant toxic effects in mice and cells and patents related to the preparation of ICA-related drugs have been published [27, 28]. Therefore, the combination of ICA and CUR is safe to some extent and these drugs are commonly used in China without imposing a significant economic burden on patients. Additionally, Moreover, gut microbiota metabolizes the active ingredients of traditional Chinese medicine, which can help maintain intestinal health by balancing the microbial population [13]. ICA-CUR exerted its action by affecting gut microbiota-SCFAs *in vivo*, rather than being an invasive treatment. Therefore, ICA-CUR possesses certain therapeutic potential, and our study may provide a new option for the treatment of PCa.

Referee 2/problem 8: It is recommended to reprocess the figures to improve clarity, ensure that all text is legible, and make color adjustments to achieve a publishable level of resolution for the figures.

In conclusion, while the current study offers significant insights into the potential therapeutic effects of ICA-CUR on prostate cancer, addressing these experimental additions and limitations could further enrich the scientific contributions and translational implications of the research.

Reply: Thank you very much for your suggestion. According to your advice, we adjusted the image pixels to 600 dpi and made color adjustments to ensure that all text is clear and legible, meeting the required resolution for publication.

**References**

1.	Cheng Y, Liu J, Ling Z: **Short-chain fatty acids-producing probiotics: A novel source of psychobiotics**. *Crit Rev Food Sci Nutr* 2022, **62**(28):7929-7959.

2.	Yuan Y, Lu L, Bo N, Chaoyue Y, Haiyang Y: **Allicin Ameliorates Intestinal Barrier Damage via Microbiota-Regulated Short-Chain Fatty Acids-TLR4/MyD88/NF-κB Cascade Response in Acrylamide-Induced Rats**. *J Agric Food Chem* 2021, **69**(43):12837-12852.

3.	Louis P, Flint HJ: **Formation of propionate and butyrate by the human colonic microbiota**. *Environ Microbiol* 2017, **19**(1):29-41.

4.	Portincasa P, Bonfrate L, Vacca M, De Angelis M, Farella I, Lanza E, Khalil M, Wang DQ, Sperandio M, Di Ciaula A: **Gut Microbiota and Short Chain Fatty Acids: Implications in Glucose Homeostasis**. *Int J Mol Sci* 2022, **23**(3).

5.	Matsushita M, Fujita K, Hayashi T, Kayama H, Motooka D, Hase H, Jingushi K, Yamamichi G, Yumiba S, Tomiyama E *et al*: **Gut Microbiota-Derived Short-Chain Fatty Acids Promote Prostate Cancer Growth via IGF1 Signaling**. *Cancer Res* 2021, **81**(15):4014-4026.

6.	Schwabe RF, Greten TF: **Gut microbiome in HCC – Mechanisms, diagnosis and therapy**. *Journal of Hepatology* 2020, **72**(2):230-238.

7.	Rizzo A, Santoni M, Mollica V, Fiorentino M, Brandi G, Massari F: **Microbiota and prostate cancer**. *Semin Cancer Biol* 2022, **86**(Pt 3):1058-1065.

8.	Zhou J, Chen Y, Wang Y, Gao X, Qu D, Liu C: **A comparative study on the metabolism of Epimedium koreanum Nakai-prenylated flavonoids in rats by an intestinal enzyme (lactase phlorizin hydrolase) and intestinal flora**. *Molecules* 2013, **19**(1):177-203.

9.	Zheng Y, Wang J, Wang J, Jiang R, Zhao T: **Gut microbiota combined with metabolomics reveal the mechanism of curcumol on liver fibrosis in mice**. *Biomed Pharmacother* 2022, **152**:113204.

10.	Wei LF, Weng XF, Huang XC, Peng YH, Guo HP, Xu YW: **IGFBP2 in cancer: Pathological role and clinical significance (Review)**. *Oncol Rep* 2021, **45**(2):427-438.

11.	Menon SS, Guruvayoorappan C, Sakthivel KM, Rasmi RR: **Ki-67 protein as a tumour proliferation marker**. *Clin Chim Acta* 2019, **491**:39-45.

12.	Li LT, Jiang G, Chen Q, Zheng JN: **Ki67 is a promising molecular target in the diagnosis of cancer (review)**. *Mol Med Rep* 2015, **11**(3):1566-1572.

13.	Feng W, Yang Z, Liu Y, Chen R, Song Z, Pan G, Zhang Y, Guo Z, Ding X, Chen L *et al*: **Gut microbiota: A new target of traditional Chinese medicine for insomnia**. *Biomed Pharmacother* 2023, **160**:114344.

14.	Sekhoacha M, Riet K, Motloung P, Gumenku L, Adegoke A, Mashele S: **Prostate Cancer Review: Genetics, Diagnosis, Treatment Options, and Alternative Approaches**. *Molecules* 2022, **27**(17).

15.	Pejčić T, Todorović Z, Đurašević S, Popović L: **Mechanisms of Prostate Cancer Cells Survival and Their Therapeutic Targeting**. *Int J Mol Sci* 2023, **24**(3).

16.	Bowling GC, Rands MG, Dobi A, Eldhose B: **Emerging Developments in ETS-Positive Prostate Cancer Therapy**. *Mol Cancer Ther* 2023, **22**(2):168-178.

17.	Pernigoni N, Guo C, Gallagher L, Yuan W, Colucci M, Troiani M, Liu L, Maraccani L, Guccini I, Migliorini D *et al*: **The potential role of the microbiota in prostate cancer pathogenesis and treatment**. *Nat Rev Urol* 2023, **20**(12):706-718.

18.	Jin D-X, He J-F, Zhang K-Q, Luo X-G, Zhang T-C: **EtOAc extract of H. attenuatum Choisy inhibits inflammation by suppressing the NF-κB and MAPK pathways and modulating the gut microbiota**. *Phytomedicine* 2019, **57**:292-304.

19.	Lu Y, Yuan X, Wang M, He Z, Li H, Wang J, Li Q: **Gut microbiota influence immunotherapy responses: mechanisms and therapeutic strategies**. *J Hematol Oncol* 2022, **15**(1):47.

20.	Koh A, De Vadder F, Kovatcheva-Datchary P, Bäckhed F: **From Dietary Fiber to Host Physiology: Short-Chain Fatty Acids as Key Bacterial Metabolites**. *Cell* 2016, **165**(6):1332-1345.

21.	Hao H, Zhang Q, Zhu H, Wen Y, Qiu D, Xiong J, Fu X, Wu Y, Meng K, Li J: **Icaritin promotes tumor T-cell infiltration and induces antitumor immunity in mice**. *Eur J Immunol* 2019, **49**(12):2235-2244.

22.	Topalian SL, Hodi FS, Brahmer JR, Gettinger SN, Smith DC, McDermott DF, Powderly JD, Carvajal RD, Sosman JA, Atkins MB *et al*: **Safety, activity, and immune correlates of anti-PD-1 antibody in cancer**. *N Engl J Med* 2012, **366**(26):2443-2454.

23.	Li T, Zhang C, Zhao G, Zhang X, Hao M, Hassan S, Zhang M, Zheng H, Yang D, Liu L *et al*: **IGFBP2 regulates PD-L1 expression by activating the EGFR-STAT3 signaling pathway in malignant melanoma**. *Cancer Lett* 2020, **477**:19-30.

24.	Voskoboinik I, Whisstock JC, Trapani JA: **Perforin and granzymes: function, dysfunction and human pathology**. *Nat Rev Immunol* 2015, **15**(6):388-400.

25.	Hervas-Stubbs S, Riezu-Boj JI, Gonzalez I, Mancheño U, Dubrot J, Azpilicueta A, Gabari I, Palazon A, Aranguren A, Ruiz J *et al*: **Effects of IFN-α as a signal-3 cytokine on human naïve and antigen-experienced CD8(+) T cells**. *Eur J Immunol* 2010, **40**(12):3389-3402.

26.	Curtsinger JM, Agarwal P, Lins DC, Mescher MF: **Autocrine IFN-γ promotes naive CD8 T cell differentiation and synergizes with IFN-α to stimulate strong function**. *J Immunol* 2012, **189**(2):659-668.

27.	**<Medical Application of Icariin in Preparing Medicament for Antagonizing Myeloid-Derived Suppressor Cells. CN. Patent No102106864 Beijing State Intellectual Property Office of the P.R.C..pdf>**.

28.	Cai F, Chen M, Zha D, Zhang P, Zhang X, Cao N, Wang J, He Y, Fan X, Zhang W *et al*: **Curcumol potentiates celecoxib-induced growth inhibition and apoptosis in human non-small cell lung cancer**. *Oncotarget* 2017, **8**(70):115526-115545.

29.	Hu J, Wu X, Yang C, Rashid K, Ma C, Hu M, Ding Q, Jiang H: **Anticancer effect of icaritin on prostate cancer via regulating miR-381-3p and its target gene UBE2C**. *Cancer Med* 2019, **8**(18):7833-7845.

30.	Sheng W, Xu W, Ding J, Li L, You X, Wu Y, He Q: **Curcumol inhibits the malignant progression of prostate cancer and regulates the PDK1/AKT/mTOR pathway by targeting miR‑9**. *Oncol Rep* 2021, **46**(5).

31.	Wei Z, Yang B, Tang T, Xiao Z, Ye F, Li X, Wu S, Huang JG, Jiang S: **Gut microbiota and risk of five common cancers: A univariable and multivariable Mendelian randomization study**. *Cancer Med* 2023, **12**(9):10393-10405.
